# A sustainable integrated agroforestry system

**DOI:** 10.3389/fpls.2025.1635422

**Published:** 2025-10-27

**Authors:** Luis R. Comolli, Esteban Schegg, Cristian Infuleski, Hugo Fassola, Alejandra von Wallis, Nardia M. Bulfe, Sara R. Barth, Paola A. Gonzalez, María Elena Gauchat, Néstor Munareto, Victoria Gross, Paula Cruz, Fabio Wyss

**Affiliations:** ^1^ El Rocio SA, Santo Pipo, Argentina; ^2^ Department of Production, Instituto Linea Cuchilla (ILC), Ruiz de Montoya, Argentina; ^3^ Department of Agricultural Extension, Asesor, Productores Yerba Mate Sociedad Cooperativa Limitada (SCL), Santo Pipo, Misiones, Argentina; ^4^ Instituto Nacional de Tecnología Agropecuaria (INTA), Estación Experimental Agropecuaria Montecarlo, Montecarlo, Misiones, Argentina; ^5^ Asesor Técnico de la Dirección General de Yerba Mate y Té, Ministerio del Agro y la Producción, Posadas, Misiones, Argentina; ^6^ School of Forest Sciences ,National University of Misiones (UnaM), Eldorado, Misiones, Argentina; ^7^ Institute of Subtropical Biology (IBS), Iguazú Node – National University of Misiones (UnaM) Consejo Nacional de Investigaciones Científicas y Técnicas -CONICET (National Scientific and Technical Research Council), Iguazu, Misiones, Argentina; ^8^ Instituto Nacional de Tecnología Agropecuaria (INTA), Centro Regional Misiones, Posadas, Misiones, Argentina

**Keywords:** agroforestry, sustainability, adaptation, mitigation, biodiversity, multi-species systems, soil restoration

## Abstract

**Introduction:**

Agricultural intensification has boosted global food supply but has also driven deforestation, soil degradation, biodiversity loss, and greenhouse gas emissions, posing critical sustainability challenges. In response, alternative practices such as agroforestry have emerged, yet few long-term experimental systems exist that integrate high biodiversity with commercial perennial crops. Over 25 years, we have developed and refined a perennial, multi-species agroforestry system in northeastern Argentina, integrating *Ilex paraguariensis* (yerba mate) and 19 associated tree species.

**Methods:**

The experimental design monitored crop yields, soil restoration metrics (including soil organic matter content), and population dynamics of the detrimental psyllid *Gyropsylla spegazziniana*. Long-term field data were collected from replicated agroforestry and monoculture plots to evaluate the ecological and agronomic performance of the system.

**Results:**

This biodiverse, perennial system restores degraded soils through sustained increases in organic matter, stabilizes or enhances *I. paraguariensis* yields relative to monoculture controls, substantially diminishes psyllid infestation through natural regulation, and facilitates broader biodiversity recovery, including avian and mammal recolonization. A striking and unplanned finding was the system’s resilience during the extreme 2021–2022 South American drought, heatwaves, and wildfires—plants within the agroforestry system showed markedly less stress than those in monocultures, underscoring its adaptive capacity.

**Discussion/Conclusion:**

These findings demonstrate the ecological and agronomic advantages of agroforestry as a scalable alternative to monoculture. By mimicking natural forests, our system shows how biodiversity-driven complexity enhances resilience, reduces dependence on external inputs, and provides climate adaptation and mitigation benefits. Integrated agroforestry offers a vital pathway to achieve the 2030 biodiversity targets of the CBD COP-15 and aligns agriculture with the UN Decade on Ecosystem Restoration.

## Introduction

**Figure 1:**
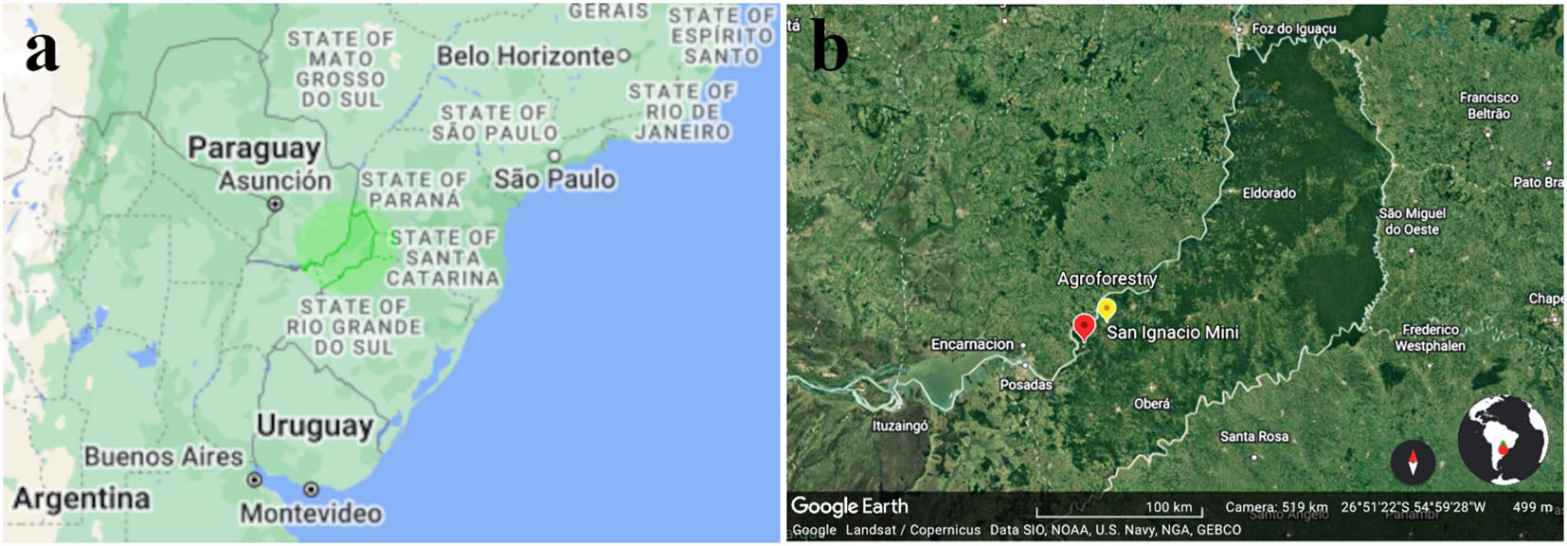
**(a)** Map of the north-east of Argentina emphasizing the province of Misiones in brighter green. Argentina, Brazil, and Paraguay are shown with present political divisions (google maps)^1^. **(b)** Magnified map^1^ of the area emphasized in **(a)**. The red “pin” marks the location of the Jesuit ruin San Ignacio Mini^2^, and the yellow one the location of the agroforestry *trial lot* where the data presented in **Tables 1** to **4** was acquired.

The Northeast of Argentina is a region that extends with the shape of a boot into the subtropical forests it shares with Paraguay to the North, Brazil to the Northeast, and Uruguay to the Southeast (**Figure 1a** (Roca, 2017; Google Maps, 2024a); **Figure 1b**, (Google Maps, 2024b)). *Ilex paraguariensis (* Heck and De Mejia, 2007; Gawron-Gzella et al., 2021), a tree species native to this region, is the source of the popular South American infusion known as “mate (Gawron-Gzella et al., 2021)”, derived from its processed dried leaves, a product referred to as “yerba mate (Heck and De Mejia, 2007; Bracesco et al., 2011; Gawron-Gzella et al., 2021; Sarreal, 2023)”. The creators of the modern version of this drink were the Jesuits (Heck and De Mejia, 2007; Bracesco et al., 2011; Gawron-Gzella et al., 2021; Sarreal, 2023; UNESCO’s World Heritage Centre, 2024) who, starting in 1609, built settlements throughout a vast territory spanning present-day Uruguay, Paraguay, Brazil, and Argentina (Roca, 2017; UNESCO’s World Heritage Centre, 2024). They refined the traditional consumption methods employed by the indigenous populations. The Jesuits domesticated the tree, established plantations, and developed the commercially viable product made drying the leaves harvested from the tree (Heck and De Mejia, 2007). In Argentina, most of the missions are in the province of Misiones (Roca, 2017; Google Maps, 2024a) (**Figure 1**). In most of Paraguay and the south of Brazil the cleared jungle was converted to arable land used for annual cereals and oilseeds production. Meanwhile in the Argentinian part of this territory patches of native forests remain healthy if not intact, mixed with planted forests, intercalated with agriculture of mostly perennial species. This fact is immediately apparent in low resolution satellite maps of the region, in which Argentina appears with a much darker green color than Brazil and Paraguay, the contrast of forests versus arable land cultivated with grains (Roca, 2017; Google Maps, 2024a) (**Figure 1b**).

Modern *I. paraguariensis*, or “yerba mate,” cultivation in northeastern Argentina has followed monoculture practices on fertile lands previously cleared of native forests. These plantations, dating back to the early 20th century, prioritized ease of management, typically employing a rectangular grid with approximately 1,110 plants per hectare, often extending across medium to large areas beyond the small farm scale of a few hectares. The advent of mechanized agriculture led to increased planting densities, often doubling to around 2,220 plants per hectare with a 1.5 x 3-meter rectangular pattern. Crop management practices, including weeding, are both labor-intensive and mechanized, typically employing tractors and mechanical plows. This modern approach, while increasing yield, replaced native forests entirely, increased reliance on external inputs, and diminished soil health and biodiversity (Gawron-Gzella et al., 2021; Sarreal, 2023).

Agrochemicals are now commonly used for weeding, reducing the reliance on plows, and for pest control. However, their widespread use has well-documented negative impacts on non-target organisms, biodiversity, and ecosystem functions, contributing to long-term environmental degradation (Gandara et al., 2024; Wan et al., 2025a; Wan et al., 2025b). These well-documented externalities further underscore the need for alternative farming systems that can reduce or eliminate reliance on many such synthetic inputs. Fertilizers are applied when economically feasible, based on local practices. The soils in this region are predominantly Ultisols (Ultisol soil) (Pereyra, 2012), characterized by high clay content and poor nutrient retention, making them heavily dependent on organic matter. After several years of mechanized crop management, these soils experience significant degradation (Pereyra, 2012). The province of Misiones, Argentina, has experienced less deforestation compared to neighboring Paraguay and Brazil (**Figure 1**), resulting in comparatively lower biodiversity loss, soil degradation, and waterway contamination. Nevertheless, preventing further land degradation remains a paramount objective, particularly as it is a more attainable goal.

This project, conceived in the early 1990s, was inspired by the recognition that *I. paraguariensis* is a forest tree, not a shrub or grass. It thrives within the complex inter-species interactions and microclimate of forests. Our approach was also informed by extensive first-hand experience and accumulated knowledge. The productivity of yerba mate plantations significantly declines as soil degrades, historically leading to further deforestation and expansion of underutilized, degraded lands. *I. paraguariensis* is also severely outcompeted by weeds and grasses, which flourish in open fields but are absent in native forests, where shrubs compete with trees for space. The cycle of deforestation for monoculture agriculture results in increased reliance on external inputs, diminished conservation of the region’s natural resources, expansion of underproductive lands (agricultural land that produces yields significantly below its potential capacity due to soil degradation) dominated by shrubs and grasses with low biodiversity, and loss of local climate regulation. Therefore, adopting attributes of the original subtropical forests appeared sensible in addressing these interconnected goals: preventing soil degradation and irreversible loss of fertility; restoring already degraded lands; maximizing the restoration of the region’s biodiversity; and establishing a viable, sustainable, and resilient long-term cultivation modality.

The primary objective in developing this integrated agroforestry system was to maximize complexity and biodiversity while achieving sustainable economic yields in *I. paraguariensis* production. Our foundational hypothesis was that this approach would facilitate the gradual attainment of restoration and conservation goals through an iterative design, calibrated by heuristically derived parameters. Increased biodiversity was expected to enhance pest control, soil physical structure and biota, soil fertility, and moisture conservation, contributing to long-term sustainability and improved local climate stability. Furthermore, we posited that a system designed for restoration, conservation, and biodiversity would yield superior long-term economic outcomes due to reduced reliance on external inputs and enhanced resilience. This approach also establishes a foundation for developing non-proprietary knowledge, technologies, and differentiated products.

In the intervening years, evidence has mounted in high-quality research confirming that biodiversity plays a fundamental role in enhancing ecosystem services and agricultural sustainability. A broad body of work demonstrates that different forms of biodiversity can enhance ecosystem services in agriculture. At the field scale, crop diversification through intercropping, cover cropping, and sown field margins has been shown to strengthen pest control, improve soil fertility, and support pollination while stabilizing yields (Wan et al., 2020b; Wan et al., 2022b; Wan et al., 2024; Wang et al., 2025). Beyond terrestrial cropping, mixed systems such as rice–fish co-culture represent powerful examples of ecological intensification, where predator–prey dynamics and nutrient cycling jointly enhance productivity and resilience (Wan et al., 2019; Wan et al., 2022a; Li et al., 2025). Similarly, vegetable–aquatic animal co-culture has been shown to provide multi-trophic benefits, increasing yields while reducing reliance on external inputs (Wan et al., 2020a). These studies collectively highlight how agrobiodiversity — whether in plant genetic diversity, multispecies cropping, or animal–plant integrations — improves ecosystem services including biological pest control, nutrient cycling, and climate regulation. Our integrated yerba mate agroforestry system belongs to this wider class of diversification strategies, specifically adapting forest complexity principles to perennial cash-crop cultivation in subtropical systems.

In place of traditional monocultures, we established an agroforestry system integrating 20 perennial species, prioritizing ecological complexity, biodiversity, and resilience. Our experimental variables, which included planned measurements, were biomass production (tree growth), soil restoration metrics (including soil organic matter content), and crop yields. We also implemented monitoring protocols for insect populations, focusing on the detrimental psyllid, *Gyropsylla* sp*egazziniana*. The integrated tree diversity supported avian populations, providing natural pest control and significantly reducing pesticide dependence, aligning with broader ecological objectives. However, the type and scale of recent climate extreme events were unforeseeable and not part of the initial research plan. Nevertheless, the system’s resilience was strikingly evident during recent historically unprecedented heatwaves, droughts, and intense rainfall, demonstrating its extraordinary capacity to buffer these extreme events.

The changing climate regime is shifting perceptions about agroforestry in the region. The severe heatwave and prolonged drought that impacted northeastern Argentina, southern Brazil, Paraguay, and Uruguay in late 2021 and early 2022 had catastrophic effects on agriculture, biodiversity, and the local economy. The region experienced significantly below-average precipitation throughout 2021, with pronounced drought periods in May-June and August-September. By the start of summer, average surface temperatures were well above historical norms, and the drought persisted until March 2022. Record-breaking temperatures, reaching 42.5 degrees Celsius on January 24, 2022 (Roset, 2022; El Territorio, 2022; Boletín climatológico, servicio meteorológico nacional, 2022), were recorded during January and February amidst extreme droughts. These extreme weather conditions, peaking between December 2021 and March 2022, resulted in widespread loss of agricultural production, young perennial species, mature forests, and wildlife. Severe, unprecedented wildfires further exacerbated these losses. This crisis fundamentally altered public perceptions regarding climate change and the advantages of agricultural practices that integrate and leverage natural ecosystem complexity in the face of climate shocks. The significance of ecosystem services became starkly apparent under these extreme conditions.

The 2021–2022 climate shock in the region underscored the importance of trees and forests, reflecting a global trend of increased extreme climate events (AghaKouchak et al., 2020; Fischer et al., 2021; Walker and Van Loon, 2023; Yuan et al., 2023; Zhou et al., 2023) that has heightened public awareness of global warming. As global temperatures rise, climate hazards are projected to increase in frequency and intensity (AghaKouchak et al., 2020). Mitigation efforts will directly impact the probability, duration, and spatial extent (Wan et al., 2024) of these record-shattering extremes (Fischer et al., 2021). With increasing global populations and urban expansion, more individuals are vulnerable to climate extremes. Furthermore, the frequency and intensity of co-occurring climate extremes, which significantly impact human welfare and ecosystem sustainability, are expected to rise (Zhou et al., 2023). For instance, simultaneous large wildfires, fueled by hot and dry conditions, can overwhelm suppression efforts, leading to extensive forest mortality and environmental damage (Zhou et al., 2023). Flash droughts, characterized by precipitation deficits and increased evapotranspiration that rapidly deplete soil moisture (Walker and Van Loon, 2023; Yuan et al., 2023), exemplify the severe impact of rapid climate shifts on ecosystems, exceeding their adaptive capacity. Higher-emission scenarios predict an increased risk of rapid-onset flash droughts, posing significant challenges for climate adaptation (Walker and Van Loon, 2023; Yuan et al., 2023).

In summary, artificial forest systems should emulate the primary direct benefits of forests, including biophysical climate regulation at local and global scales, atmospheric humidity regulation, and the provision of habitat that supports biodiversity and ecosystem services (Feng et al., 2022a; Gurevitch, 2022; Feng et al., 2022a; Meyfroidt et al., 2022). The UN Decade on Ecosystem Restoration (UN Environment Program, 2023) emphasizes that “ecosystems support all life on Earth” (UN Environment Program, 2023). Restoration can be achieved through active planting and the removal of stressors to facilitate natural recovery. However, returning ecosystems to their original state is often neither feasible nor desirable (Meyfroidt et al., 2022; UN Environment Program, 2023). We must accommodate existing land use, such as agriculture and infrastructure, and acknowledge that ecosystems, like societies, must adapt to a changing climate (Our World in Data, 2023; World Resources Institute, 2023).

This paper documents the scalable cultivation of a subtropical agricultural species at high density within a managed, diverse forest system, contrasting with traditional monocultures. Our collaborations with public extension agencies have facilitated capacity building and public outreach, demonstrating the economic benefits of ecological restoration. We advocate for agroforestry, as defined by the Food and Agriculture Organization of the UN (FAO) (FAO, 2023), and similar initiatives as crucial strategies for achieving the 2030 biodiversity targets outlined in the Global Biodiversity Framework of the CBD COP-15.

## Methods

### Experimental design

**Figure 2:**
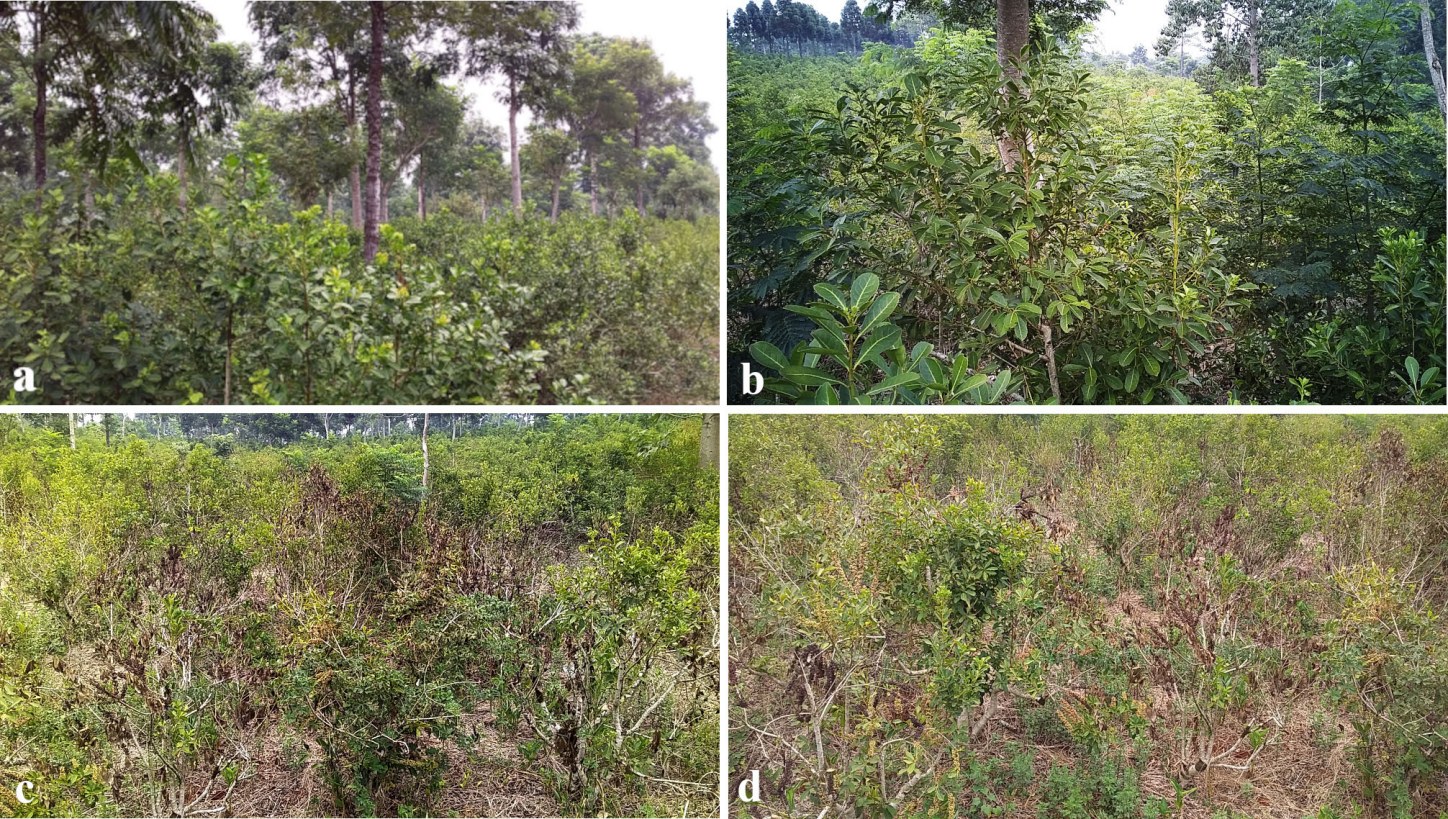
A comparison between yerba mate plants consociated with trees, **(a, b)**, and a control with plants in a monoculture, without intercalated trees, **(c, d)**, during the extreme climate shock of 2022. **(a)** is a lot of 10 ha planted in 2011, and **(b-d)** show views within the agroforestry experimental *trial lot* of 10 ha, all on the same soil with the same history. **(b)** shows a close-up view of yerba mate plants in close association with trees, to be compared with **(c, d)**, the control lot without trees immediately adjacent to that shown in **(a)**. The brown color in **(c, d)** are dried leaves and branches. Photos taken in January 2022. Cadaster details for all images in all figures are provided in section Experimental Context of the **Supplementary Information**.

**Figure 3:**
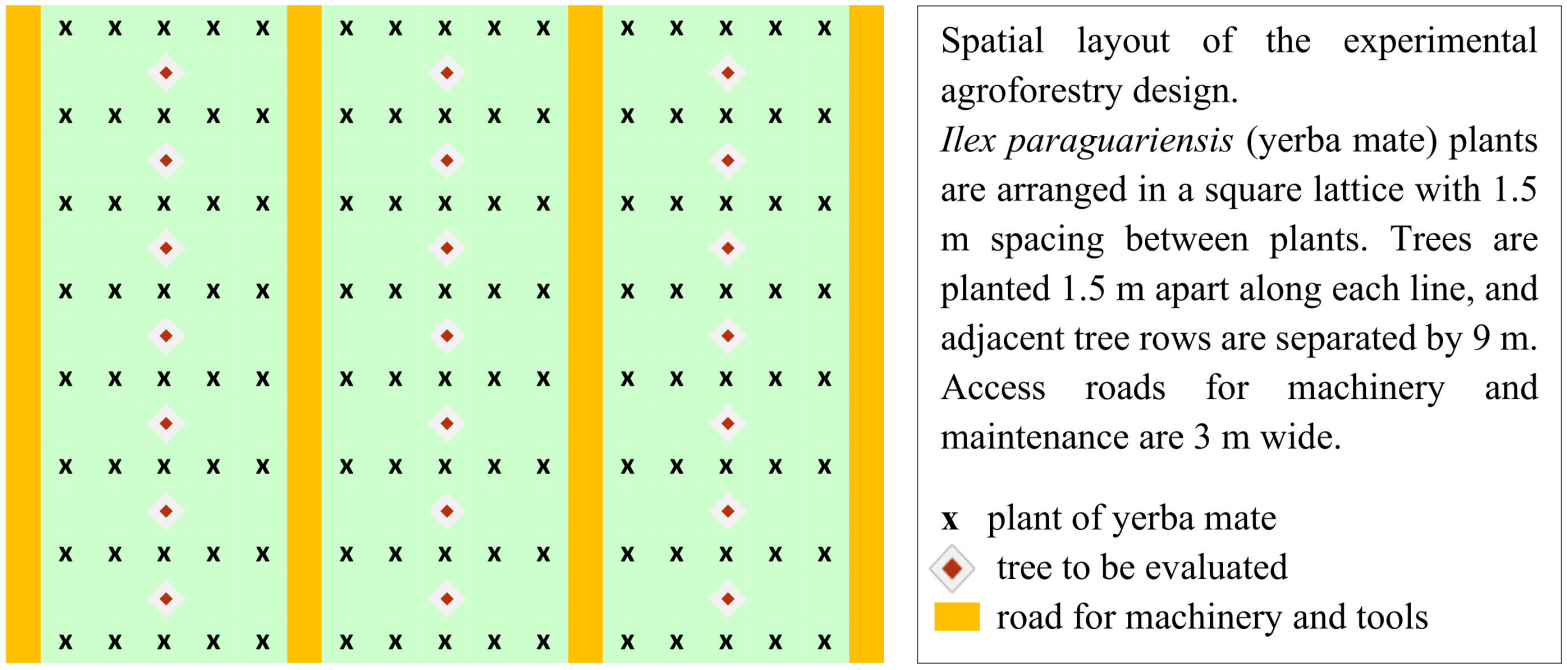
Arrangement of *I. paraguariensis* and trees within the agroforestry system.

In 2010 we established an experimental agroforestry “*trial lot”* with nine species of trees (**Table 1**) consociated with I. paraguariensis (yerba mate, **Figures 1**–**3**), spanning an area of 10 ha, on soil cleared from an artificial pine forest installed approximately 15 years earlier for fallowing of old yerba mate plantations (schematic in **Supplementary Figure 1**). This experimental lot is identified as Lots 1 and 3 within “Lote XII” (approximately 98 ha), cadaster of the municipality of Santo Pipo, Misiones, Argentina (https://earth.google.com/ 27° 8’34.77”S, 55°23’37.18”W). Each species, chosen to represent a range of native and exotic options with perceived or potential benefits for yerba mate cultivation, covers four randomized sublots with a combined surface area of 1 ha per species. Four sub-lots totaling 1 ha were left free of trees by design as control for future measurements and direct visual comparisons (the initial implementation has been described by Comolli et al (Comolli et al., 2024)). Although *I. paraguariensis* is a full-size tree, in yerba mate cultivation it is trimmed to the size of a large shrub for practical reasons (**Figure 2a**; **Supplementary Figure 2**). This experimental lot was designed to quantify a collection of anecdotal evidence on the favorable consociation of *I. paraguariensis* with several native tree species, in another lot of 10 ha implanted years before, 2003, as a heuristic pilot project (**Supplementary Figure 2d**).

**Table 1:** Tree species being experimentally tested in the multispecies agroforestry system.

English name	Scientific name
Lapacho negro*	*Handroanthus heptaphyllus*
Lapachillo	*Handroanthus pulcherrimus*
Lapacho amarillo	*Handroanthus albus*
Petiribi (loro negro)*	*Cordia trichotoma*
Loro blanco	*Bastardiopsis densiflora*
Cedro Misionero (Cedar)	*Cedrela fissilis*
Araucaria*	*Araucaria angustifolia*
Cañafístola*	*Peltophorum dubium*
Anchico Colorado*	*Parapiptadenia rigida*
Guatambú*	*Balfourodendron riedelianum*
Guayubira	*Cordia americana (ex Patagonula americana)*
Tipa	*Tipuana tipu*
Urunday	*Astronium fraxinifolium (Astronium balansae)*
Curupay	*Anadenanthera colubrina*
Toona (Australian cedar)*	*Toona ciliata*
Grevillea*	*Grevillea robusta*
Kiri*	*Paulownia* sp
Hybrid pine	Hybrid pine F1 (*Pinus elliottis & Pinus caribaea hondurensis*)
Taeda pine or loblolly pine	*Pinus taeda*

Species with (*) are those in the 10 ha *trial lot* planted in 2010, from where measurements presented in **Tables 2**–**4** were obtained. The species listed cover over 200 ha of *I. paraguariensis* agroforestry in various distributions.

Subsequently, these experimental trials were expanded to include a total of 19 tree species (**Table 1**), now spanning over 200 hectares, following a heuristic approach. While the scale of system precludes routine scientific data acquisition across all variables beyond the designated “*trial lot”*, it serves as a valuable large-scale control, underpinning comparative studies for each variable at chosen time points, and the testing of new species and hypotheses.

The ability to measure self-consistent data sets from the *trial lot* is enabling meaningful comparisons between species and, as the global climate changes, to re-examine choices of species and the parameters and timing of tree management practices such as trimming and thinning and regrowth. This controlled experimental design allows for annual measurements of various parameters, including yerba mate yield, insect populations, and soil characteristics, for each tree species ‘treatment’ and the control, providing a basis for direct comparisons. All the consolidated data we present in **Tables 2**–**4** of this manuscript correspond to the experimental variables planned for this *trial lot*. The full scale of the agroforestry project adequately enables further field experimentation for each conclusion derived from the *trial lot*. We have avoided compositionally simple tree planting and continue to explore new varieties to add to the mix. The goal is to find the largest possible number of species with synergies, to achieve the greatest diversity. The current pace of climate change and the magnitude of extreme climate events were not anticipated during the project’s implementation. Consequently, we did not design pre-defined experimental variables to specifically investigate their impact (IMRAD structure style). However, given their significant relevance, we include comparative observations of their effects on the trial lot and across the broader 200-hectare system. More details about the region and its climate, fine-grained contextual information, and geography, in Comolli et al (Comolli et al., 2024) and **Supplementary Information**.

**Table 2:** Yerba mate harvest production over 8 years.

Species/year	2014	2015	2016	2017	2018	2019	2020	2021	2022	Total/species	Err
Anchico	1221	4835	4238	3979	4288	6021	5080	8205	7703	45569	21
Toona	1807	6987	5939	5131	4341	5659	6731	9275	6927	52797	23
Guatambu	1132	4568	4213	4283	4361	4871	6038	7854	6680	44000	21
Araucaria	1405	5460	6120	5579	5746	5719	6825	9928	8964	55745	24
Lapacho	1375	5469	5817	4738	4919	5636	5506	10901	9430	53792	23
Loro Negro	1478	5839	5317	4873	5649	5745	5001	8703	7325	49931	22
Grevillea	1272	4760	5437	4282	4365	5452	6269	8738	7409	47985	22
Caña Fistula	1544	5182	5263	4020	4577	5759	5943	8456	6521	47264	22
Control	1235	4823	5016	5214	5246	6029	4812	8834	7203	48412	22
Kiri	1523	5828	5369	4570	4124	6442	4772	10175	8599	51402	23
Total/Year	13993	5375	23808	4667	4762	5733	5697	9107	7676	496896	70

Harvest of *I. paraguariensis* in kilograms per ha per species of consociated tree and for test lots, integrated over the 10 ha *trial lot*, from 2014 to 2022. Err is the propagated error from the systematic repetitive weight measurements (Methods).

**Table 3:** Comparative count of *Gyropsylla* sp*egazziniana* insects in 2017.

Date	Sample	Agroforestry	Control (no trees)
31/08	1	5	4
13/09	2	16	22
20/09	3	65	63
03/10	4	27	39
11/10	5	19	28
18/10	6	11	96
27/10	7	136	412
2/11	8	308	613
9/11	9	292	549
15/11	10	102	194
24/11	11	43	70
6/12	12	29	22
15/12	13	18	5
22/12	14	15	2

Comparative counts of insects for the spring season of one chosen year. Number of psilido (*G.* sp*egazziniana*) individual insects counted in traps (Methods and Supplementary Discussion) within the agroforestry *trial lots* in comparison with standard monoculture lots without trees, with date of sampling as “day/month”. The estimated error per counting event, represented with the error bars in **Figure 6**, is less than 10%, Methods and Supplementary Discussion.

**Table 4:** Soil test results for the agroforestry *trial lot* over an interval of 8 years.

Year	OOM%	TOM %	ROOC %	P2O5 ppm
2013	2.39 ± 0.56	3.12 ± 0.72	1.39 ± 0.32	7.26 ± 5.36
2019	1.99 ± 0.2	2.59 ± 0.3	1.16 ± 0.1	10.54 ± 1
2021	2.55± 0.43	3.34 ± 0.56	1.49 ± 0.25	12.72 ± 7.87

Average values with the standard deviation of the mean for: Oxidizable Organic Matter, OOM%; Total Organic Matter, TOM%; Readily Oxidizable Carbon, ROC %; Extractable Phosphorous, P2O5 (ppm). These values result from averaging 145 species-specific soil test results. OOM and TOM are derived from ROOC, they are reported here for the general reader (see also **Supplementary Tables 1A, B** in **Supplementary Discussion**). See Methods and **Supplementary Information** for details in a broader context.

### Plants, establishment density, and evolution

**Figure 4:**
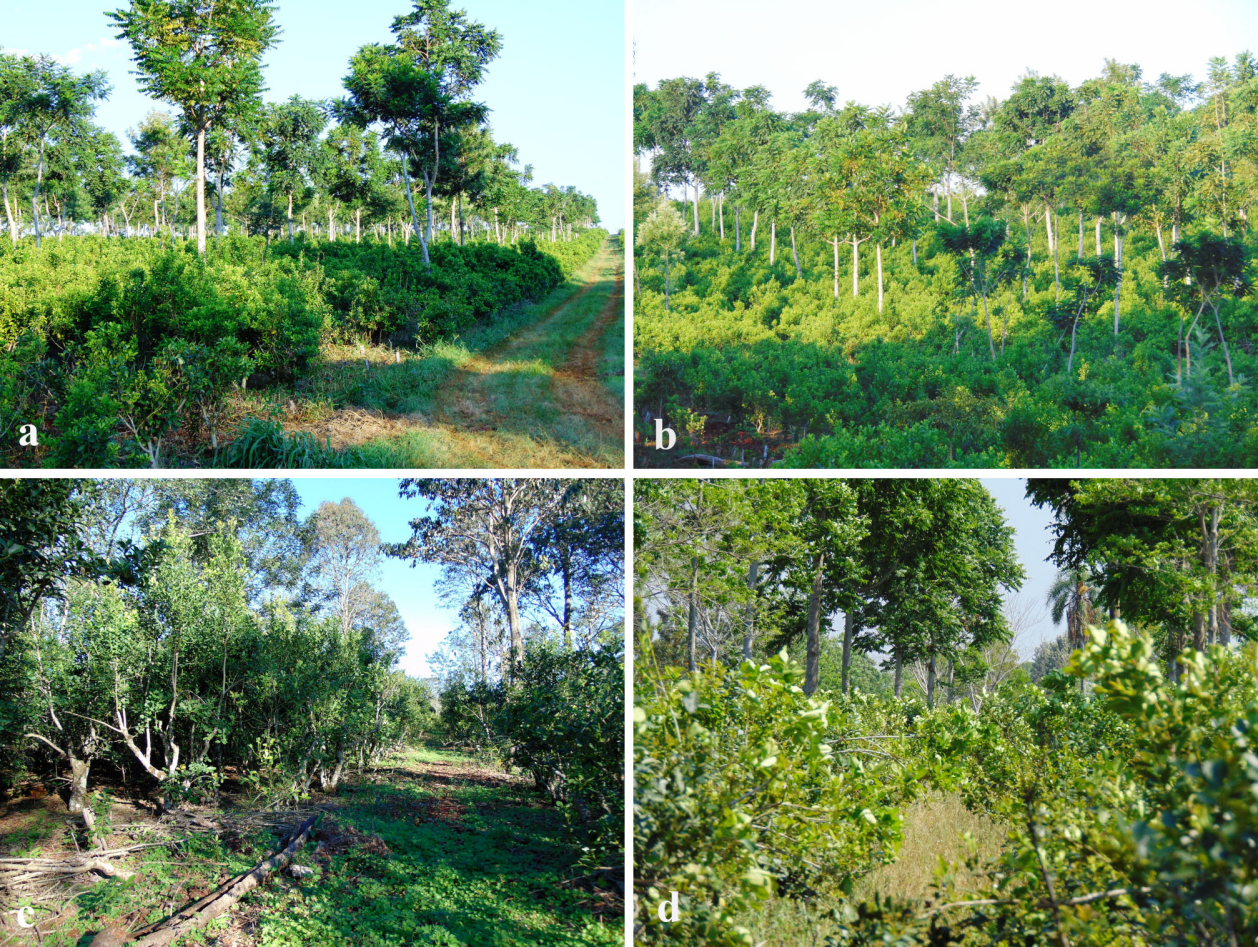
Overview of the agroforestry system before the climate shock. **(a, b)** show views of the same lot presented in **Figure 2a** but approximately 4 years earlier, in 2018. The trees had been trimmed of the lower branches. **(c)**, a view of a lot planted in 2005, showing trimmed branches left on the ground. Photo taken in 2017. **(d)** is a view of the experimental pilot lot shown in **Figure 2**, from a different observation point, showing ryegrass as a ground cover where sun exposure allows it to grow. Photo taken in 2017. The density is of approximately 3,700 *I. paraguariensis* (mate) plants (see also **Supplementary Figure 2a**) and 246 trees per hectare.

*I. paraguariensis* seedlings used in this study were derived from the seeds created at “Estación Experimental Agropecuaria Cerro Azul” INTA (EEA Cerro Azul), Ruta Nacional 14 Km 836, Cerro Azul, Misiones, Argentina. The seedlings of Toona, Grevillea, and Kiri, were obtained from local seed orchards, who practice selective breeding to enhance specific traits, ensuring a degree of genetic advancement. The seedlings for native species were made in-house from seeds collected from trees within the remnants of native forests in the local region, meticulously selected based on their phenotypic characteristics such as crown size and height. The spacing was 1.5 meters between plants of yerba mate (*I. paraguariensis*) in 5 adjacent lines or rows separated by 1.5 meters (**Figure 3**). These groups of 5 lines are separated by 3 meters, leaving space for machinery. Initially the density was approximately 3,700 *I. paraguariensis* plants and 740 trees per hectare, intercalated with the central line of *I. paraguariensis* plants at a distance of 1.5 meters between trees (Zhou et al., 2023) (see **Figures 2a**, **3**, **4**). After five years the trees were thinned down to approximately 246 trees per ha for each species, and after eight years to 123 trees per ha. The total area for each species and controls, divided in four samples or sublots, is 1 ha. Therefore, the measured production of mate is by construction the production per ha.

The selection of tree species for our agroforestry systems has evolved over time, informed by both observational experiences and a desire to maximize ecological and productive synergies with yerba mate. The initial selection, from 2003 to 2010, was guided by anecdotal evidence suggesting beneficial consociation between yerba mate and specific native species such as Cañafístola, Araucaria, Lapacho, and Cedar (as seen in **Supplementary Figure 2d** and **Supplementary Figure 4**). This initial palette was subsequently expanded to include other species, such as the native legume Anchico, known for its potential to improve soil nitrogen, and the exotic Toona (a type of cedar), which also showed promising early interactions. Our long-term experience also includes working with various pine species, particularly loblolly pine (*Pinus Taeda*, **Supplementary Figure 7f**). The nine species planted in the *trial lot* described above were chosen to represent a range of native and exotic options with perceived or potential benefits for yerba mate cultivation. They provide the measurements of our experimental variables and guide our ongoing effort to improve the system as climate change aggravates.

### Image data

The photographs shown in figures were acquired with a digital camera Sony Cyber-shot 20.1 Mega Pixels, DSC-H300 and with a cell phone Samsung Galaxy J4 SM-J410G. Images documenting small mammals, presented in **Supplementary Figure 8**, were acquired with a camera trap Browning BTC-5DCL, located off-road and attached to the base of a yerba mate plant, at a height of about 30 cm.

### Harvest volume measurements

**Figure 5:**
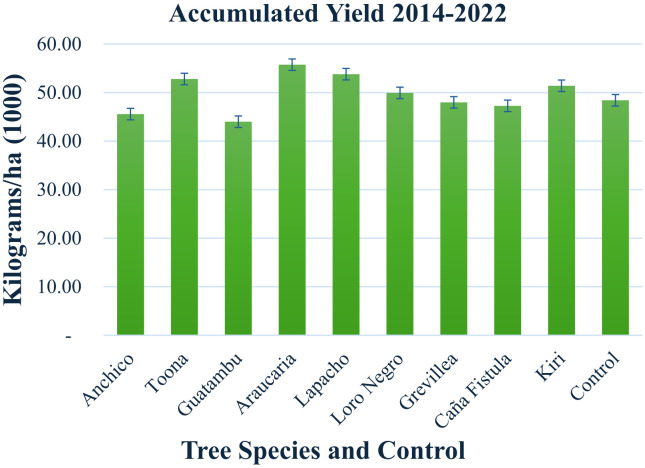
Histogram of the total accumulated yield of yerba mate per tree species per ha presented in **Table 2**. The scale is expressed in 1,000 kgs. Each specie and control span a 1 ha surface, giving yield per ha automatically. The number of plants is approximately 3700 yerba mate plants and 246 adult trees per ha years 2015-2018, 123 trees per ha after 2019. A graph showing results per tree species and per year is presented in **Supplementary Figure 9**. The error bars represent the propagated error for the systematic weight measurements (Methods).

The harvest of yerba mate is done manually, with each person first gathering the harvest in packages of approximately 100 kilos. The weight of each package is measured with a portable scale (a pylon balance or an electronic pylon balance -Hook Scale) with an uncertainty of 0.1 kg and collected in specially prepared truck chassis, then transported to the roaster and dryer facility (a volume of 10,000 kg results from 100 measurements with errors normally distributed, so the propagated uncertainty is $\sqrt{N}\;\times \;$ 1 kg = $\sqrt{100}$ × 1 kg  =10 kg (1)) . For these volumes the propagated errors are in a range of values several orders of magnitude smaller than the sum of values and are not visible nor clearly meaningful in the chart. The magnitude of interest is the total harvest, which is a sum of values. The display in **Figure 5** has error bars approximately 10 times the values obtained propagating the uncertainty or error over 400 to 550 weight added measurements to be safe, ultra-conservative, and to aid in visual display. There is no meaning to averages (i.e. per mate plant) in this context. Additional contextual and historical details in **Supplementary Discussion**.

### Soil tests (soil compositional analysis)

In Spanish we use “Ensayo de Suelo”; these are effectively Soil Compositional Analysis. Over 156 standardized soil test samples were obtained during the period 2010-2022. Samples were collected from each location using a 0.25 m ring sampler at 0.2 m of depth (volume 1 L), temporarily stored in 2 Kg commercial plastic bags, and transported to the laboratory. Each sample is a composite of six subsamples. The method used for estimations of soil organic carbon (ROC %) is based by the chromic acid titration method by Walkley and Black (Walkley and Black, 1934), standardized in Argentina by the Instituto Argentino de Normalización y Certificación (IRAM) with norm “IRAM-SAGyP 29571-3:2016 Parte 3: Determinación de Carbono orgánico oxidable por mezcla oxidante fuerte- microescala” (Secretaria de Agricultura and Ganaderia y Pesca, 2024). Oxydazable Organic Matter (OOM) and Total Organic Matter (TOM) are obtained from ROC by multiplicative factors that depend on the context. The instrumental uncertainty for soil tests values is approximately 9%. The values produced by these standardized soil compositional analysis tests are shown in **Supplementary Tables 1A, B**. The values reported in **Table 4** were computed averaging 145 individual samples (107 samples for 2013, 36 samples for 2021, and 6 samples binned in two aggregated samples for 2019) using the statistics functions in Excell, with a custom-made Python code, and with a custom-made Wolfram language code (available upon request) to cross-validate the results. See the Appendix of the Supplementary Information with a detailed example. All soil tests analysis, including **Tables 4**, **Supplementary Tables 1A, B**, were carried out at the Instituto Nacional de Tecnología Agropecuaria (INTA), Estación Experimental Agropecuaria Cerro Azul (EEA Cerro Azul), Ruta Nacional 14. Km. 836, Cerro Azul, Misiones C. P. (3313). Additional contextual and historical details in **Supplementary Discussion**.

### Counts of insects in traps

The number of “psilido” (psyllid *Gyropsylla* sp*egazziniana*) individuals were counted using Moericke traps within the agroforestry *trial lots* and in standard monoculture lots without trees. The traps were “home-made”, using commercial Tupperware as a box, with water, and an inverted beer bottle to replenish the water as it evaporates (**Supplementary Figure 10**). Trapped insects were manually counted by Engineer Nestor Munareto, and randomly chosen bins were counted again by Engineer Cristian Infuleski typically 3 more times to estimate the counting error. The error is estimated to be well below 10% of the count for each counting event. This estimated human counting error derived by the repeated independent counts of randomly chosen bins approximately follows a normal distribution. Insect counts labelled as “counts with trees” were done in the agroforestry pilot or test lot identified as Lots 1 and 3, within “Lote XII”, cadaster of the municipality of Santo Pipo, Misiones, Argentina. Insect counts labelled as “counts without trees” were done in Lot 13, within “Lote XII” (https://earth.google.com/ 27° 8’26”S, 55°23’55”W), adjacent to the agroforestry *trial lots*.

No chemical insecticides were used during the entire period reported here.

### Statistical methods

Statistical results including means, standard deviations, error propagation, and statistical significant tests, were computed using the statistics functions in Excell, with a custom-made Python code, and with a custom-made Wolfram language code (both available upon request) to cross-validate the results.

### Supplementary discussion (measurements in context and experimental context)

The Supplementary Information uploaded along the main text include a full description of the Experimental Design, extensive fine-grained contextual information on Materials and Methods, and Geographical Location of each source of data as well as additional contextual visual information. Scientists and agronomists interested in details of the extensive field work may find useful the context provided in the Supplementary Discussion of the Supplementary Information, subtitled measurements in context and experimental context.

## Results

### Climate mitigation

The extreme drought and heat wave of 2021–2022 provided a direct visual perception of some of the benefits afforded by trees such as shade protection, humidity conservation, and modulation of the local temperature. The salient observations are the drying up of plants and the hotter microclimate in large areas without trees, aspects further elaborated in the Discussion section. Yerba mate cultivated in lots with trees fared the drought and extreme heat considerably better at first sight, **Figure 2**. **Figure 2a** shows the mate plants, pruned to the size of large shrubs and grown in consociation with trees, with healthy aspect, while **Figure 2b** shows a zoom-in view of the state of yerba mate plants consociated with trees. In contrast, plants left in open sky, not consociated with trees, show the impact of the harsh climate. **Figure 2c** shows the control without trees, adjacent to 2b, under visible stress, while **Figure 2d** shows a close-up view of yerba mate plants in this control without trees (a few meters to the left of 2b); they are dying, the brown color corresponds to dead branches and leaves.

Plants in the control sublots devoid of associated trees (**Supplementary Figure 1**), clearly demonstrate markedly elevated stress levels. For a comprehensive visual comparison, refer to **Supplementary Figures 2a, b**, which illustrate both a high-density and a conventional yerba mate lot as monocultures prior to the climate shock. Additionally, **Figure 4** and **Supplementary Figures 2c, d** present images of agroforestry lots before the climatic event. **Supplementary Figures 4** and **5** provides additional examples of the visible stress in plants without the mitigating protection afforded by trees in contrast to plants consociated with them. The yerba mate plant density in these agroforestry lots ranges from 3,200 to 3,700 plants per hectare, while the initial density of trees is approximately 741 trees per ha in all lots the first 5 years. These are thinned to 246 trees per ha until year 8, and finally 123 trees and 123 pruned regrowing shoots after 9 years. (details in Supplementary Information). High-density lots initially contained 3,700 mate plants per ha without trees (**Supplementary Figure 2a**), which were planted several years after the yerba mate. These tree densities provide the mitigation effect shown in these images.

### Harvest yields

Productivity, soil development, and natural insect population control were the key metrics for comparing yerba mate performance in monoculture and agroforestry systems. **Table 2** and **Figure 5** provide a comparative analysis of yerba mate harvest yields per hectare for each tree species in trial (with trees) and control lots (without trees) from 2014 to 2022. The histogram in **Figure 5** illustrates the cumulative yield for each species and the control over the entire period. The time course of these yields can be found in **Supplementary Figure 9** and **Supplementary Discussion** (Measurements in Context).

Yerba mate harvest yields are higher with five species of consociated trees compared to control lots without trees, as detailed in **Table 2** and the histogram in **Figure 5**. The yield variations are within 13% or less. Notably, the native species Guatambu exhibited approximately 10% lower yields than the control, while Araucaria and Lapacho, also native species, exhibited approximately 13% and 10% higher yields than the control, respectively (these differences are several times the uncertainty in the measurement of total yields, see methods). Additionally, two exotic species, Toona and Grevillea, demonstrate higher yields compared to the control. Due to its excessive size for the management of this project, Kiri was phased out of the system by natural extinction, so the yields represent open sky with decomposing organic matter.

It’s noteworthy that the observation period coincides with the development of the system, from the planting of seedlings to the growth of adult trees. The most productive tree associations varied over the years, as indicated in **Supplementary Figure 9**. The first harvest season after the climate shock, the fall and winter of 2022 showed an overall decrease in production, as evidenced in **Table 2** and **Figure 5**, and further detailed in **Supplementary Figure 9**.

A critical factor in our agroforestry system is adult tree density, which varies in time based on the tree species, desired coverage and space competition, and the feasible scale of cultural work (cultivation practices) maintenance. Experiments with plastic shade meshes indicated optimal yerba mate harvest yields at 30% to 40% shade compared to full sunlight. Therefore, based on these findings, we have manually trimmed the trees for the first 10 years of each planted lot to prevent excessive light blockage (see **Supplementary Figure 5d**). The trimmed branches are left on the ground to decompose and enrich the soil with organic matter (as shown in **Figure 4c**, **Supplementary Figures 5b**, **6b**).

### Biological diversity

**Figure 6:**
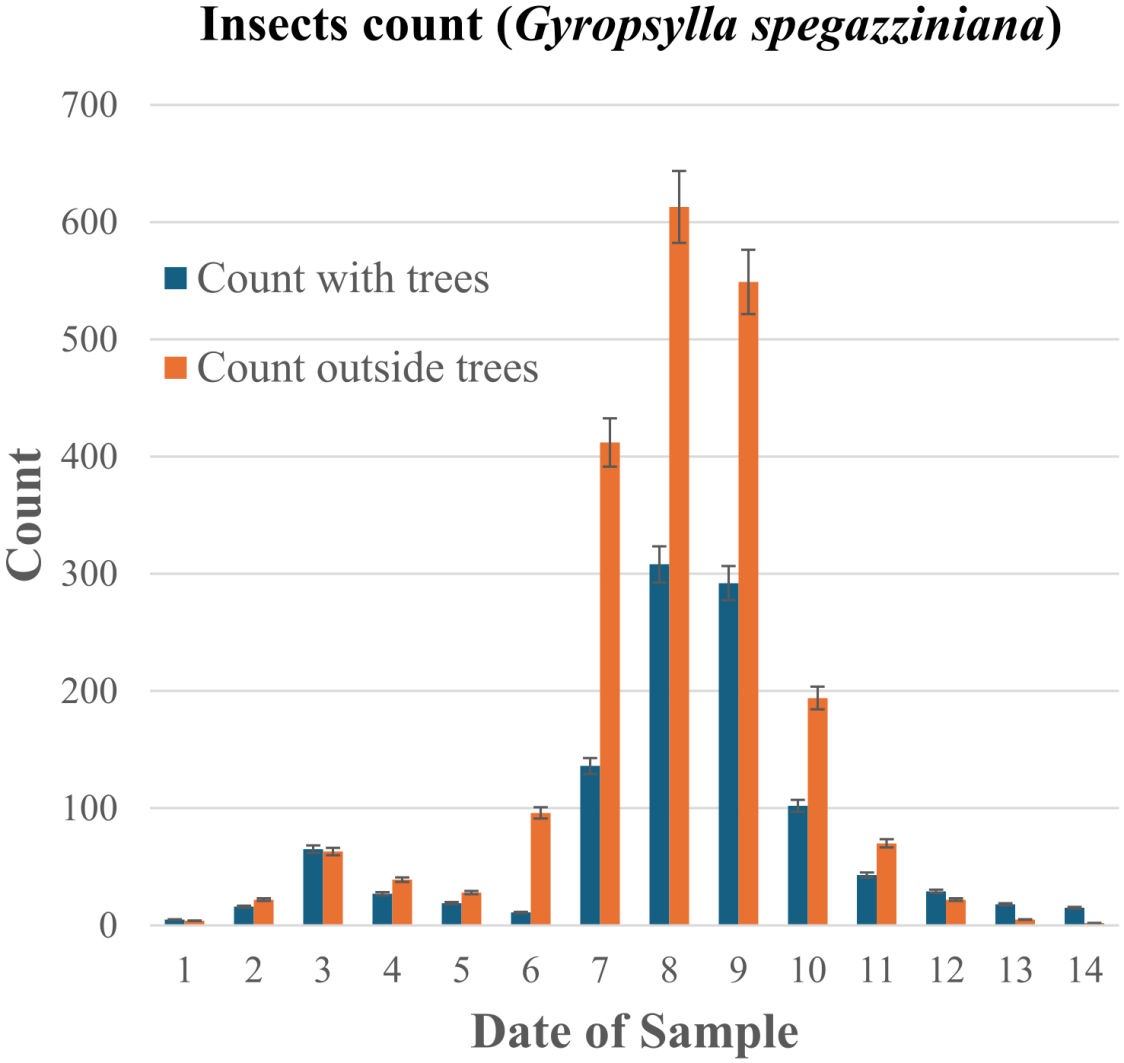
Histogram of the data presented in **Table 3**. The estimated error per counting event, represented with the error bars, is less than 10%, see Methods and Supplementary Discussion. The complete data set, which includes other years and another agroforestry project, is being processed and analyzed (S.R.B, work in progress), and will be published in a more specialized journal on this subject.

At 8 to 10 years of age, tree density was reduced by fifty percent, managing regrowth and replacing any mortalities. While the wood could be used commercially, we choose to add it to the soil to further enhance its quality (refer to **Figure 4c**; **Supplementary Figure 6b**). The current tree density and this management support a bird population (illustrated in **Supplementary Figures 5c**, **6f**, and **7b**), which helps control harmful insects, consequently reducing the need for broad-spectrum insecticide applications that can negatively impact non-target wildlife. Insecticide application was only occasional, typically in response to peaks of external influxes from neighboring areas. **Table 3** and the histogram in **Figure 6** compare the number of a specific detrimental insect, the ‘psilido’ or *Gyropsylla* sp*egazziniana*, found during a key period in the agroforestry *trial lot* with those found in two neighboring monoculture control lots (without trees) at the same time. At the peak of population counts, the control monoculture lot had twice the counts, with a difference many times the uncertainty.

We also consider the variation in ground cover, specifically grasses, and the associated maintenance costs of weeding. In our agroforestry system, the dense planting of *I. paraguariensis* and trees results in a winter ground cover of ‘docile’ herbs (illustrated in **Figure 4c**; **Supplementary Figures 5b**, **5d**, **5e**). In spring, the ground is generally covered with planted and naturally regrown ryegrass (see **Figure 4d**; **Supplementary Figures 5e**, **6d**, **6e**). However, we observe that as the trees mature, the extent of ground cover decreases (as shown in **Supplementary Figure 6e**). During the establishment phase of plantations, we cover the ground with legumes in the summer months (depicted in **Supplementary Figure 6c**) and with ryegrass during winter and spring (**Supplementary Figures 6d, e**). This strategy of ground cover management aligns with the different growth stages of the trees and *I. paraguariensis*, optimizing soil health and reducing the need for external maintenance. Consequently, we observed an increase in overall biodiversity within the agroforestry system.

The combination of these elements has resulted in the return of a range of small aboriginal mammals, typical to the region, as documented in a collaboration with the Institute of Subtropical Biology (IBS, Iguazú node – National University of Misiones (UnaM)- Consejo Nacional de Investigaciones Científicas y Técnicas -CONICET (National Scientific and Technical Research Council of Argentina)), shown in **Supplementary Figure 8**.

### Soil organic matter

To assess and monitor soil quality, we regularly conduct soil tests. These tests track key parameters, including organic matter content, soil acidity, and aluminum levels, the latter being an indicator of soil acidification.

**Figure 7:**
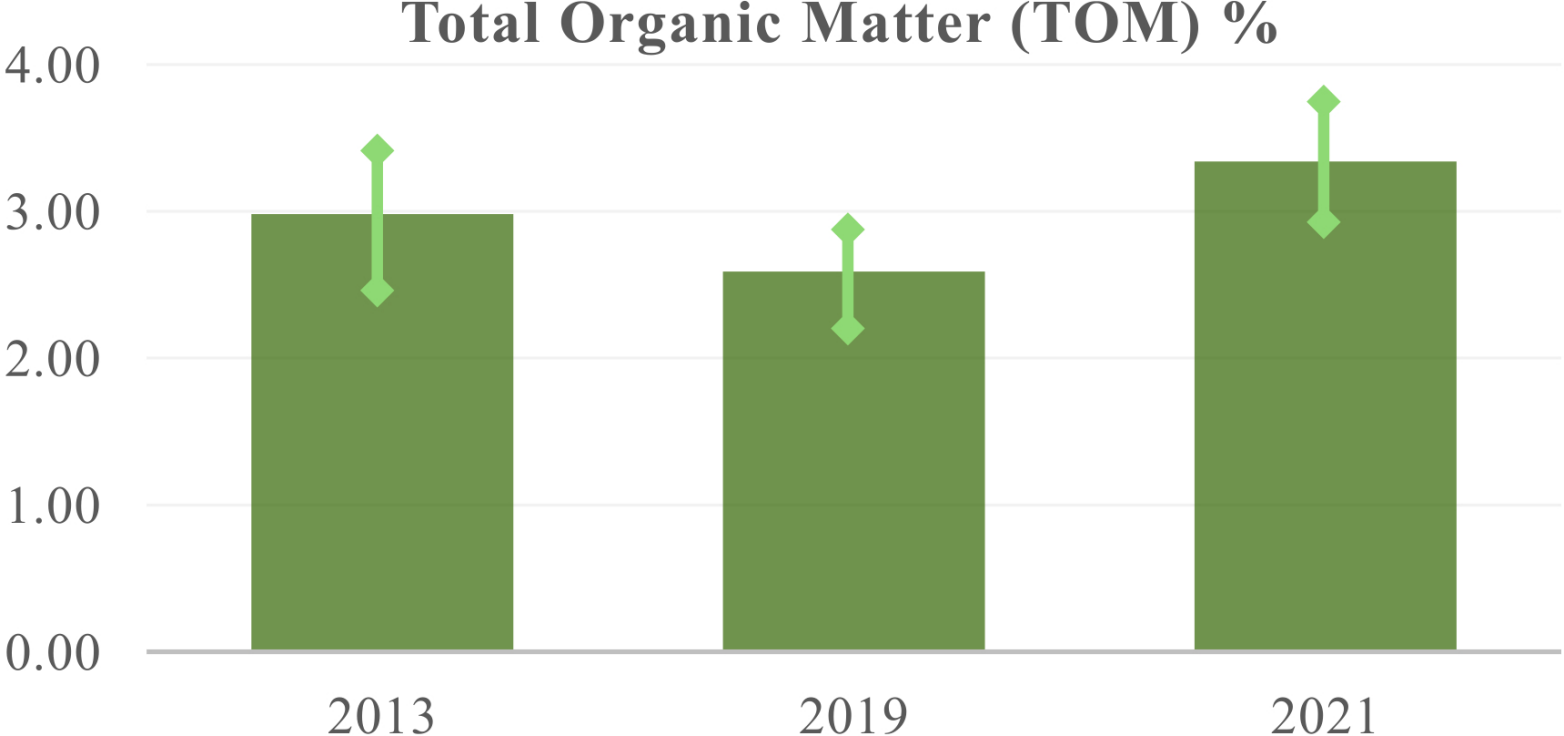
Total Organic Matter, TOM% for years 2013, 2019, and 2021 presented in **Table 4**. The error bars represent the standard deviation of the mean as reported in the table. See Methods and Supplementary Discussion for more details.

A notable result is the observed increase in soil organic matter, a contrast to the typical decrease seen in monoculture cultivation with tillage following deforestation. After an initial decrease, we’ve noted a rising trend in organic matter accumulation, achieving levels of over 0.5% per year (as shown in **Table 4**; **Figure 7**). The difference between the average TOM for 2021 and 2013 has a p-value of 0.07, indicating a statistical significance of 0.07. While this p-value is greater than the standard significance level of 0.05, the relevance of the result is that there is a recovery with an upward slope for TOM values. It is also important to note that soil samples taken up until 2021 do not fully reflect the decomposition of trimmed branches and cut trees into the soil (see **Figure 4c** and **Supplementary Figure 6b**). As the evolution of TOM values is a direct result of the management of the growing trees (growing biomass), future stronger results are to be expected. Soil tests also highlight the varying contributions of different tree species, a result that is context dependent. An example is shown in the Appendix of the Supplementary Information. While these specific contributions are aggregated in this table to show the overall impact, there are species-specific variations (work in progress, A. von W., N.M.B).

**Supplementary Information Tables 1A** and **1B** provide an example of a comparison of soil test results for *I. paraguariensis* monocultures from neighboring lots with those from our agroforestry system. These compositional soil analyses are done routinely across the extended system. Even at these early stages, our agroforestry system shows better organic matter content compared to the reference monoculture lots. The monoculture lots exhibit worse acidity, with lower base saturation levels (a measure of the soil’s capacity to exchange cations) and significantly higher aluminum levels, which are toxic for farming. More recent soil analyses indicate continuous improvement in these critical parameters. However, this gradual improvement underscores the difficulty in reaching these results and importance of preventing soil degradation in the first place, a significant result itself.

A significant result that was not part of the plan or the objectives has been created or at least enhanced by the severity of current climate shocks. Trees in our agroforestry system create a microenvironment that not only fosters a greater diversity of animal life but also provides a more comfortable working environment. They offer protection from the harsh sun in summer, reduce the severity of cold in winter, and lessen the impact of heavy rains. The management of the system creates a range of tasks that expands the know-how, and the skill set required from workers (as shown in **Supplementary Figure 7**). Such an environment inherently adds resilience and robustness to the system, which has an impact on biodiversity, TOM, and harvest yields. The diversity of activities also leads to a variety of synergistic work tasks the reinforce mitigation and adaptation, fostering a sense of shared objectives (illustrated in **Supplementary Figures 7c-7e**) which result in an iterative self-reinforced feedback loop strengthening the results listed above throughout the years.

### Our three approaches to establishing agroforestry

To overcome the limitations of monoculture systems (**Supplementary Figures 2a**, **b**), we implemented and observed three primary approaches to establishing integrated agroforestry systems: artificial forest conversion, direct high-density multi-species planting, and ecological succession enrichment. In all cases we verify that the incorporation of multiple tree species integrated with the harvest species enhances the adaptability and resilience of the plantation in the face of extreme climate events. A detailed discussion is presented in the Supplementary Information.

## Discussion

Our comparative analysis of a long-term integrated agroforestry system for yerba mate cultivation and traditional monocultures revealed several significant outcomes. Firstly, the agroforestry system effectively halted soil degradation and initiated a notable recovery in soil organic matter content. Secondly, we observed higher yerba mate yields per hectare when cultivated in consociation with specific tree species compared to monoculture controls. Thirdly, the population of the detrimental insect *Gyropsylla* sp*egazziniana* was significantly reduced within the agroforestry plots. Furthermore, the established biodiverse environment, integrating multiple perennial plant species– a significant contribution to biodiversity – also facilitated the return of native small mammals and enhanced the overall resilience of the system to extreme climate events. Finally, our observations underscore the substantial potential of agroforestry as a strategy for both climate adaptation and mitigation in agricultural landscapes.

The observed increase in soil organic matter (SOM) within the agroforestry system represents a significant finding, particularly when contrasted with the soil degradation typically associated with *I. paraguariensis* monoculture agriculture and tillage (Pereyra, 2012). Our results, detailed in **Table 4** and **Figure 7**, demonstrate a clear upward trend in SOM accumulation following an initial period. The difference in average TOM (Total Organic Matter) between 2021 and 2013 evidences a net recovery, characterized by a consistent upward slope indicative of soil restoration.

This trend is particularly noteworthy given that soil samples taken up to 2021 do not fully account for the contribution of decomposing trimmed branches and cut trees, a key management practice in our system (**Figure 4c**; **Supplementary Figure 6b**). We anticipate that future soil analyses, incorporating these factors, will reveal even more pronounced improvements in soil organic matter.

We also measure various agricultural indicators (e.g. **Supplementary Table 1**). Fertilizers are applied periodically, which introduces a complex bias in the nitrogen, potassium, and phosphorus levels (standard commercial triple nitrogen (N), phosphorus (P) and potassium (K) (NPK) at the rate of 50 kg per 1,000 kg of harvested yerba mate distributed uniformly across whole lots. Monitoring phosphorus, a limited natural resource essential for plant growth is particularly crucial, as it is not only tied to organic matter levels but also becomes less accessible to plants in acidic conditions.

The observed increase in SOM has several important implications. Higher SOM levels improve soil structure, enhancing water retention and reducing erosion. They also increase nutrient availability and support a thriving soil biota, contributing to long-term soil health and fertility. This contrasts sharply with monoculture systems, where tillage and the lack of organic matter inputs often lead to soil compaction, erosion, and nutrient depletion. The varying contributions of different tree species to SOM will be explored in future work. However, the overall trend demonstrates the potential of agroforestry to reverse soil degradation and promote soil restoration, reduce the need for external inputs, and enhance overall soil fertility, crucial steps towards sustainable agriculture.

Our research demonstrates a notable enhancement in yerba mate harvest yields when cultivated in association with five specific tree species compared to monoculture controls lacking tree cover (**Table 2**; **Figure 5**). The observed yield variations remained within a modest range of 13% or less, suggesting that the integration of these particular tree species does not impose a substantial negative impact on overall productivity, and in several cases, even enhances it.

Interestingly, the native species *Guatambu* (*Balfourodendron riedelianum*) exhibited approximately 10% lower yields than the control. In contrast, two other native species, *Araucaria angustifolia* and *Lapacho* (*Handroanthus impetiginosus)*, showed promising results with approximately 13% and 10% higher yields than the control, respectively. These yield differences are statistically significant, exceeding the measurement uncertainty for total yields (as detailed in the Methods section). Furthermore, the exotic species *Toona ciliata* and *Grevillea robusta* also demonstrated higher yields when consociated with yerba mate. The case of *Paulownia tomentosa* (Kiri), which was phased out due to its growth characteristics, indicates that its presence likely transitioned the understory to open sky conditions with increased organic matter from its decomposition, potentially influencing yields during its presence.

These findings directly challenge the conventional assumption that tree shade and competition invariably lead to reduced yerba mate yields. They are also consistent with work showing much greater response ratios for artificial forests made of a variety of different species than mono species (Hua et.al.) (Hua et al., 2022). Our results suggest a more nuanced interaction, where specific tree species can create synergistic interactions (such as microclimate regulation, potential improvements in soil health, and indirect pest control) that are beneficial for yerba mate productivity. This enhanced productivity has significant implications for the economic viability and attractiveness of adopting agroforestry practices for yerba mate cultivation, offering a pathway to both ecological sustainability and increased agricultural output.

A significant ecological benefit observed in our agroforestry system is the substantial reduction in the population of the detrimental insect, the psyllid (*Gyropsylla* sp*egazziniana*), a key pest in *I. paraguariensis* cultivation. As detailed in **Table 3** and **Figure 6**, the agroforestry trial lots exhibited a twofold decrease in the peak population counts of this insect compared to monoculture control lots monitored concurrently. This difference was statistically significant, exceeding the measurement uncertainty by several orders of magnitude. These findings suggest that the increased structural and biological complexity of the agroforestry system creates an environment less favorable for this specific pest.

The presence of a diverse avian population within the agroforestry system, supported by the integrated tree structure (**Supplementary Figures 5c**, **6f**, **7b**), likely plays a crucial role in this natural pest control. The multi-layered canopy and permanent tree cover provide perching sites, nesting opportunities, and sheltered microhabitats that attract and sustain insectivorous birds. From these vantage points, birds can forage efficiently, patrolling the understory and feeding on sap-sucking insects such as psyllids. This creates a direct top-down regulatory effect on *G.* sp*egazziniana* populations eliminating the need for chemical insecticides. Furthermore, the increased diversity of plant life could support a more balanced community of insects, including beneficial species that may compete with or prey on *G.* sp*egazziniana*. This demonstrates how structural complexity in agroforestry systems can be harnessed as a nature-based solution for pest regulation, reducing dependence on synthetic inputs while simultaneously restoring ecological functions.

The reduced psyllid populations in the agroforestry system have several positive implications. Firstly, they directly contribute to healthier *I. paraguariensis* plants, minimizing the damage caused by this sap-sucking insect and potentially leading to more stable and higher yields. Secondly, this natural pest control mechanism reduces the reliance on synthetic insecticides, mitigating potential negative impacts on non-target organisms, soil health, and the overall biodiversity of the agroecosystem. This finding underscores the potential of agroforestry to promote a healthier ecosystem and sustainable pest management strategies.

Beyond our initial objectives focused on soil and productivity, the long-term establishment of a perennial, multi-species agroforestry system has yielded a notable increase in biodiversity. The integration of twenty perennial plant species, comprising a mix of native and exotic trees alongside *I. paraguariensis*, has created a more complex and structurally diverse habitat compared to the simplified environment of monoculture plantations (**Supplementary Figures 2a**, **b**).

This enhanced structural complexity provides a wider array of niches, supporting a greater abundance and diversity of life across different trophic levels. Notably, our collaborative work with the Institute of Subtropical Biology (IBS-CONICET) documented the return of several species of small aboriginal mammals, typical to the region, within the agroforestry plots (**Supplementary Figure 8**). This rewilding of native fauna is a strong indicator of the system’s capacity to restore ecological functions and connectivity within the landscape. The presence of these native species is a key indicator of improved ecosystem health and a greater degree of ecological balance. Several factors likely contribute to this increase in biodiversity. The trees provide varied habitats and shelter, while the diverse plant community offers a wider range of food sources and niches. Additionally, the reduced need for broad-spectrum insecticides in the agroforestry system allows for the survival and proliferation of a wider array of insect species, which in turn support other trophic levels. The enhancement of biodiversity within agricultural landscapes offers numerous ecological benefits, including improved soil health, enhanced nutrient cycling, and increased resilience to environmental perturbations. This self-regulating capacity contributes to the overall stability and resilience of the agroecosystem, reducing the need for external interventions and promoting a more naturally balanced environment.

A striking and unplanned outcome of our long-term study was the remarkable resilience exhibited by the integrated agroforestry system in the face of the historically unprecedented heatwaves, droughts, and intense rainfall experienced in the region during 2021-2022. The evolution of global climate and these types of events are clearly outside any possible IMRAD-structured research plan. Visual evidence (**Figures 2**, **4**; **Supplementary Figures 3**, **4**) clearly demonstrated significantly less stress in *I. paraguariensis* plants grown in close proximity to various tree species compared to those in open monoculture conditions.

Several interacting mechanisms likely contributed to this enhanced resilience. The shade provided by the tree canopy effectively reduced direct solar radiation and moderated surface temperatures, while the presence of trees also contributed to local humidity conservation and potentially improved soil moisture retention through reduced evaporation and enhanced infiltration. These factors effectively reduced evapotranspiration and prevented excessive heat stress in the *I. paraguariensis* plants. The presence of a diverse root system within the agroforestry plots, including both trees and *I. paraguariensis*, likely improved water infiltration and retention, enhancing the system’s ability to withstand prolonged drought periods. Additionally, carbon sequestration within the soil matrix retains humidity. Furthermore, the overall microclimate regulation provided by the trees, including increased humidity and moderated temperatures, contributed to a more stable and less stressful environment for the understory *I. paraguariensis*.

The system’s design also allows for ongoing ecological enrichment. This multi-species approach is consistent with research (e.g. Feng et al (Feng et al., 2022a)), which demonstrates that multi-species forest plantations surpass monocultures in several key aspects, including water retention, erosion control, wood production, and biodiversity enhancement. These principles of biodiversity and ecological interactions have been present in many traditional agricultural practices.

For instance, throughout the southern region of Brazil that was part of the Jesuit settlements smallholder’s yerba mate production remained, to a considerable extent, an agroforestry crop legacy from the aboriginal practices. As noted by Lacerda (2023), traditional yerba mate production in the Araucaria Forest is intrinsically a biodiverse system, where silvicultural practices go beyond mere crop cultivation. These practices encompass an in-depth understanding of forest structure, tree species demography and diversity, and ecological succession (Lacerda, 2023). Farmers use this knowledge to sustainably manage the forest itself in the context of yerba-mate production (Lacerda, 2023). Much of this wisdom is rooted in traditional and Indigenous knowledge, built around the fact that *I. paraguariensis* is a native tree species in the forests of this region, producing foliage in the shade of consociated tree species by nature (Lacerda, 2023).

This observed resilience underscores a critical advantage of agroforestry systems in the context of increasing climate variability and extreme weather events. The observed resilience to extreme heat and drought demonstrates a clear adaptive advantage over monoculture systems, which are often severely impacted by such events. Notably, this pattern of resilience was observed across all three agroforestry establishment methods we employed, highlighting the robustness of the integrated approach. The success of our agroforestry system in mitigating these impacts suggests a powerful strategy for climate adaptation and mitigation.

Moreover, the biophysical effects of trees within agroforestry systems, including albedo modification, increased surface roughness, and regulation of evapotranspiration and moisture balance (Wilson and Lovell, 2016; Lawrence et al., 2022; FAO, 2023), contribute to local and potentially regional climate regulation. By mimicking the structure and function of natural forests, agroforestry can enhance the overall environmental benefits of agricultural lands, moving beyond a purely extractive model towards a more sustainable and ecologically integrated approach (van Noordwijk, 2021; van Noordwijk et al., 2021; IPBES, 2016).

Furthermore, the inherent characteristics of agroforestry systems contribute to climate change mitigation globally. The integration of a diverse array of tree species facilitates significant carbon sequestration both aboveground (in tree biomass) and belowground (in enhanced soil organic matter). As noted by several studies (Wilson and Lovell, 2016; FAO, 2023; Lacerda, 2023), forests and trees are well-established carbon sinks, and their incorporation into agricultural lands can play a crucial role in drawing down atmospheric CO2 (IPCC, 2023). Forests also contribute to the atmospheric balance by releasing oxygen, ozone, methane, and other gases (IPCC, 2023). Additionally, they directly influence climate through a range of biophysical and biogeochemical effects (Wilson and Lovell, 2016; Lawrence et al., 2022; FAO, 2023). These include the albedo effect, surface roughness, regulation of evaporation and moisture balance, and surface temperature moderation.

Our observed increase in soil organic matter accumulation *in situ* further supports this mitigation potential. While quantitative assessments of carbon sequestration within our specific system are ongoing (S. R. B., work in progress), the demonstrated increase in biomass and soil carbon suggests a significant contribution to climate change mitigation. Our findings align with the broader potential of agroforestry to offer local, market-driven solutions for addressing critical aspects of global climate change by strategically integrating essential characteristics of forests into agricultural practices.

The diverse management practices inherent in our integrated agroforestry system expand the know-how and skill set required of agricultural workers (**Supplementary Figure 7**), fostering a more adaptable and resilient workforce. This multifaceted work environment, characterized by synergistic tasks and a sense of shared objectives attending to climate mitigation and adaptation (**Supplementary Figures 7c-e**), contributes to the long-term sustainability and robustness of the system, indirectly reinforcing the observed benefits in biodiversity, soil organic matter, and harvest yields.

The multifaceted benefits of forest ecosystems, as recently summarized by Gurevitch (2022), include the modulation of local climates, retention of rainfall and moderation of water runoff, soil conservation, carbon sequestration, and support for biodiversity (Gurevitch, 2022). This understanding underscores the critical importance of forest systems as models for land restoration, soil erosion prevention in agriculture, and as a blueprint for developing effective strategies to adapt to and mitigate global climate change. Emulating these natural mechanisms is pivotal in addressing environmental challenges.

However, current approaches to biodiversity and ecosystem services often face limitations, hindering their effectiveness in reversing ecological decline and its impacts on human well-being (NCP) (FAO, 2023; Our World in Data, 2023; UN Environment Program, 2023; World Resources Institute, 2023). Effective ecosystem management is a complex challenge (a wicked problem) (DeFries and Nagendra, 2017), poorly addressed by simplistic, linear, top-down strategies. Rather than pursuing narrowly defined, idealized scenarios prone to implementation failures, practical compromises that garner broad acceptance are preferable, facilitating incremental yet conceptually sound advancements. Furthermore, context-specific solutions can generate positive cascading effects in surrounding areas.

The increasing recognition of agroforestry’s multifaceted role in addressing climate change challenges, as highlighted in numerous scholarly works (Fernandez et al., 1997; Montagnini et al., 2006; Verchot et al., 2007; Day et al., 2011; Frison et al., 2011; Montagnini et al., 2011; Beddington et al., 2012; Muschler, 2016; Wilson and Lovell, 2016; Capellari et al., 2017; van Noordwijk, 2021; van Noordwijk et al., 2021; Lawrence et al., 2022; Lacerda, 2023; IPBES, 2016), strongly supports our findings. Our integrated system demonstrates that agroforestry not only provides a pathway to restore soil fertility and enhance agricultural biodiversity and food security but also serves as a critical link in both adaptation and mitigation strategies against global climate shifts, a point also emphasized by the Intergovernmental Panel on Climate Change (IPCC) (IPCC, 2016). The observed resilience to extreme weather events in our study directly exemplifies this adaptive capacity.

The site-specific implementation of our integrated agroforestry system, tailored to the unique geographical, geological, and climatic factors of northeastern Argentina, underscores the importance of context-dependent approaches in sustainable agriculture. Our iterative management strategy, grounded in identifying and utilizing empirical regularities, has allowed for the continuous development and refinement of effective methods for achieving biodiversity and sustainability within our specific environment. This adaptive approach, particularly crucial in the face of dynamic climate change, aligns with and complements progressive forest management strategies observed globally (Wilson and Lovell, 2016; van Noordwijk, 2021; van Noordwijk et al., 2021; Gurevitch, 2022; Lawrence et al., 2022; Lacerda, 2023; IPBES, 2016). By contributing to the understanding and implementation of such practices, we aim to support a broader movement towards more enlightened and ecologically sound land use based on the unprecedented level of science and technology available today (International Institute for Applied Systems Analysis, 2019).

## Conclusions

In conclusion, our long-term investigation into an integrated multi-species agroforestry system for yerba mate cultivation provides compelling evidence for its multifaceted benefits, extending beyond the initial objectives of soil restoration and sustainable productivity to encompass significant climate change adaptation and mitigation potential. Our integrated yerba mate agroforestry system belongs to a wider class of diversification strategies and is fully consistent with the aims and results to date of sustainable intensification work (Pretty, 2018; MacLaren et al., 2022; Paut et al., 2024; Yu et al., 2025). Furthermore, it specifically extends the principle of ecological complexity into the perennial, tree-based domain. By embedding a commercial understory crop within a long-lived, multispecies forest scaffold, it demonstrates how the biodiversity benefits observed in annual and aquatic systems (Wan et al., 2019; Wan et al., 2020b; Wan et al., 2020a; Wan et al., 2022b; Wan et al., 2022a; Wan et al., 2024; Li et al., 2025; Wang et al., 2025) can be translated into resilient, perennial agroecosystems capable of delivering both ecological restoration and sustained productivity (Paut et al., 2024; Rivest et al., 2025; Yu et al., 2025).

The interconnectedness of enhanced soil health, increased biodiversity, stable yields, and remarkable resilience to extreme climate events underscores the ecological and economic advantages of this approach over traditional monocultures. Notably, beyond these direct ecological and economic impacts, the system has also fostered the well-being and personal development of those involved in its daily activities, aligning with objectives of the UN Decade on Ecosystem Restoration (UN Environment Program, 2023) and the Nature Futures scenarios initiative (IPBES) (Gurevitch, 2022), highlighting the broader human capital benefits of agroforestry (Rockström et al., 2017; Kremen and Merenlender, 2018; Pörtner et al., 2023). A substantial part of the costs of conventional agriculture are borne collectively as externalities (Pörtner et al., 2023; Elouafi, 2024; Gandara et al., 2024; Wan et al., 2025a; Wan et al., 2025b; Yu et al., 2025) —indeed, in large part as a global tragedy of the commons (Ostrom et al., 1999).

The demonstrated capacity of this system to foster natural pest control, improve soil fertility, and buffer against environmental shocks positions it as a scalable and adaptable model for sustainable agriculture in subtropical regions and beyond. This extensive and methodical experience, consistent with previous research on the value of native tree species in complementing agriculture (Montagnini et al., 2006; Wilson and Lovell, 2016; International Institute for Applied Systems Analysis, 2019; van Noordwijk, 2021; van Noordwijk et al., 2021; Feng et al., 2022b; Gurevitch, 2022; Hua et al., 2022; Lawrence et al., 2022; Meyfroidt et al., 2022; Lacerda, 2023; Our World in Data, 2023; Pörtner et al., 2023; IPBES, 2016; UN Environment Program, 2023; World Resources Institute, 2023; Paut et al., 2024; Rivest et al., 2025), suggests that the fundamental variables and concepts underpinning our integrated agroforestry system can be adapted and applied to various agricultural activities globally, potentially leveraging the opportunities presented by the digital revolution (International Institute for Applied Systems Analysis, 2019) for transferable strategies in sustainable land use.

## Data Availability

The raw data supporting the conclusions of this article will be made available by the authors, without undue reservation.
