# Supplementary material for: A sustainable integrated agroforestry system
Source: Front Plant Sci. 2025 Oct 27;16:1635422. doi: 10.3389/fpls.2025.1635422 (PMC12598783; doi:10.3389/fpls.2025.1635422)
Supplement: Supplementary file 1 [file DataSheet1.pdf]

Supplementary Information

Table of Contents

Experimental Design and Timeline 1
Planted density of yerba mate and of trees. 2
Supplementary Figures, Tables, and Graphs 3
Pathways to Sustainable Agroforestry Development 6
Documentation of Everyday Life and Biodiversity in this Agroforestry System 8
Microclimate Regulation 12
Supplementary Discussion 13
Measurements in context 13
Experimental Context 15

Experimental Design and Timeline

The development of our agroforestry project was a gradual process that began in the 1990s. Initially, we targeted a 40-hectare area of extremely degraded yerba mate plantations, established in 1934, and converted it into single-species pine (*Pinus elliottis*) artificial forests. This conversion was aimed at soil restoration through fallowing, with the expectation that the land could later be redeveloped into yerba mate plantations incorporating tree species.

The first significant step in our project involved an 11-hectare lot (referenced in Figure 3 c), Supplementary Figure 2 d), and Supplementary Figure 4), where *Ilex paraguariensis* (yerba mate) was implanted while preserving the regrown native tree species. This lot was further enriched with additional native tree species like Cañafístola, Petiribi, and Lapacho. This initial foray into integrated agroforestry provided valuable insights and led to the establishment of a 10-hectare agroforestry pilot or "trial lot" from the initially restored 40 hectares (detailed in Supplementary Figure 1). This pilot project was methodically laid out to facilitate comparative analysis between various tree species and control lots that were devoid of trees.

Following the encouraging outcomes from the pilot phase, we expanded this agroforestry model to additional areas of the previously restored land. Over time, this expansion led to the current state of the project, which now encompasses approximately 200 hectares of *Ilex paraguariensis* agroforestry. This progressive scaling is documented in various figures and Supplementary Information material, including Figure 3, Supplementary Figure 2 c), Supplementary Figures 5, 6, and 7 a), b), and e)). Throughout this process, our approach was driven by the aim of creating a sustainable and biodiverse agroforestry system that would not only rehabilitate the degraded soil but also enhance the productivity and ecological value of the land.

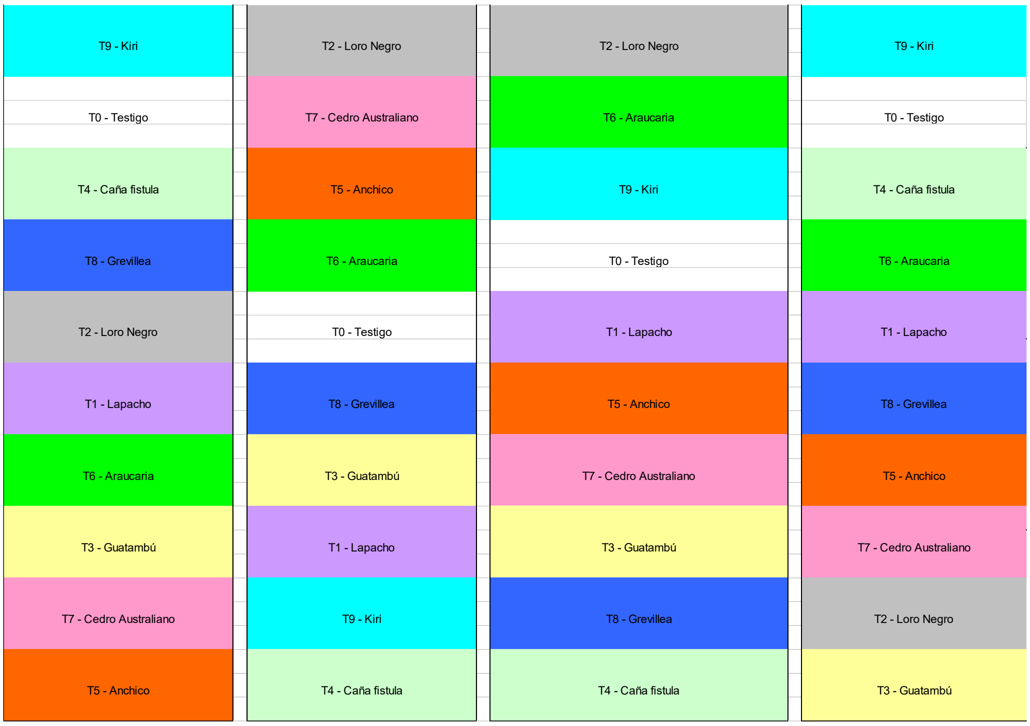

Supplementary Figure 1. Design of the 10 has agroforestry trial lot planted in 2010 with 9 tree species and a control free of trees ("testigo"). The tree species are listed in main manuscript Table 1, and are colour coded for clarity. The control lots free of trees and background are white, the tree species are coloured to evidence their randomized distribution spanning 4 blocks. The design of our 10-hectare agroforestry trial lot, established in 2010, incorporated a variety of nine tree species alongside a control section without trees (referred to as "testigo" in the chart, which means control). The layout and management of this lot were meticulously planned to provide a robust framework for evaluating the impact of different tree species on yerba mate production and other ecological factors. Initially the density was approximately 3,700 *I. paraguariensis* plants and 740 trees per hectare. After five years the trees were thinned down to approximately 246 trees per ha for each species, and after eight years to 123 trees per ha. The total area for each species and controls, divided in four samples or sublots, is 1 ha. The production of mate is by construction the production per ha. This "trial lot" spans lots 1 and 3 (Google Earth, https://earth.google.com/ 27° 8'30.47"S, 55°23'32.72"W and 27° 8'34.77"S, 55°23'37.18"W respectively), within "Lote XII" (~98 has), cadastre of the municipality of Santo Pipo, Misiones, Argentina. Referred to as the "trial lot" in this report, it serves as the primary site for gathering critical data. Measurements taken from this lot include yerba mate production per year, insect counts, and soil analysis. The goal is to assess the productivity of yerba mate under different tree species and compare it with the control plot.

Planted density of yerba mate and of trees.

All *I. paraguariensis* seedlings derived from the seeds created at Estación Experimental Agropecuaria Cerro Azul INTA (EEA Cerro Azul), Ruta Nacional 14 Km 836, Cerro Azul, Misiones C.P. (3313), Argentina. The seedlings of Toona, Grevillea, and Kiri, were obtained from local seed orchards, who practice selective breeding to enhance specific traits, ensuring a degree of genetic advancement. The seedlings for native species were made in-house from seeds collected from trees within the remnants of native forests in the local region, meticulously selected based on their phenotypic characteristics such as crown size and height. The planting strategy in our agroforestry lots was meticulously designed for optimal interaction between yerba mate (*Ilex paraguariensis*) and

various tree species. The initial planting density and subsequent adjustments were based on empirical observations and aimed at achieving the best possible synergy between the components of the system.

The spacing was 1.5 meters between plants of yerba mate (*I. paraguariensis*) in 5 adjacent lines or rows separated by 1.5 meters, making for paired 5-fold lines of mate plants. We call these rows "paired" or "composite" mate rows. These are spaced by 3 meters between them, leaving space for "farm roads" for machinery. The architecture in groups of 5 adjacent rows separated by a space of 3 meters results in 3700 plants per ha. The trees were planted in one row at the centre of each composite row, with 1.5 meters of separation between trees (of a given species for a given group of neighbouring rows). The rows of trees were thus separated by 9 meters, which results in 740 trees per ha. The trees were subsequently decimated to a space of 4.5 meters between trees within each row as their growth relative to *I. paraguariensis* exceeded expectations. Tree trunks and branches were cut down "in-situ", and regrowth is being managed. This thinning brought the density of trees to approximately 246 trees per ha and provided valuable material to the soil. Today the design is 4 paired rows of yerba mate spaced at 1.5 meters, with 3 meters separating each composite row, and one row of trees spaced at 4.5 meters from each other at the centre. This design results in 3500 plants of *I. paraguariensis* and 246 trees per ha. At an age of 7 to 8 years half the remaining adult trees are cut down and left to decompose in the grown, bringing the number of trees to 123 per ha. The regrowth is managed, and any dead tree replanted, so that when the new trees reach a good size, the older trees are cut down in a cyclical fashion, ensuring a continuous presence of young trees and decomposing wood on the ground.

Supplementary Figures, Tables, and Graphs

Examples of high-density and "modern traditional" plantations of *I. paraguariensis* are shown in Supplementary Figure 2. The plantation shown in Supplementary Figure 2 a) was planted in 2006 with a density of 3,700 plants of *I. paraguariensis* per ha on restored soil. This is the standard density used in this agroforestry project, shown here without trees for added clarity. The image in Supplementary Figure 2 b) is the most common modern type of plantation, with plants spaced by 1.5 m along rows separated by 3 m, which results in a density of 2,200 plants per ha. Most plantations done on cleared forests since the 1980s are of this type, while older plantations were done on a grid of 3 m for a density of 1,100 plants per ha. This lot had two previous cycles of perennials, tung trees (*Vernicia fordii*) and chinaberry trees (*Melia azedarach*). The lot shown in Supplementary Figure 2 a) had been initially planted in 1934 on virgin land cleared of the forest, with plants spaced at 3 m, i.e. one thousand plants per ha. By 1990 it had approximately 250 plants per ha and the land was eroded, so it produced approximately 10% of the original yield. The lot was converted to an artificial pine forest which stood for 14 years and was subsequently reconverted to high-density *I. paraguariensis* in 2006, with trees added ten years later. Supplementary Figures 2 c) and d) shows images of high-density mate plantations consociated with trees. The lot in panel c) was planted with *I. paraguariensis* and trees in 2014 directly on an old plantation dating from 1934, which was eliminated, without a period of fallowing. The lot in panel d) was planted on an abandoned orange tree plantation respecting and enriching the native trees that were present.

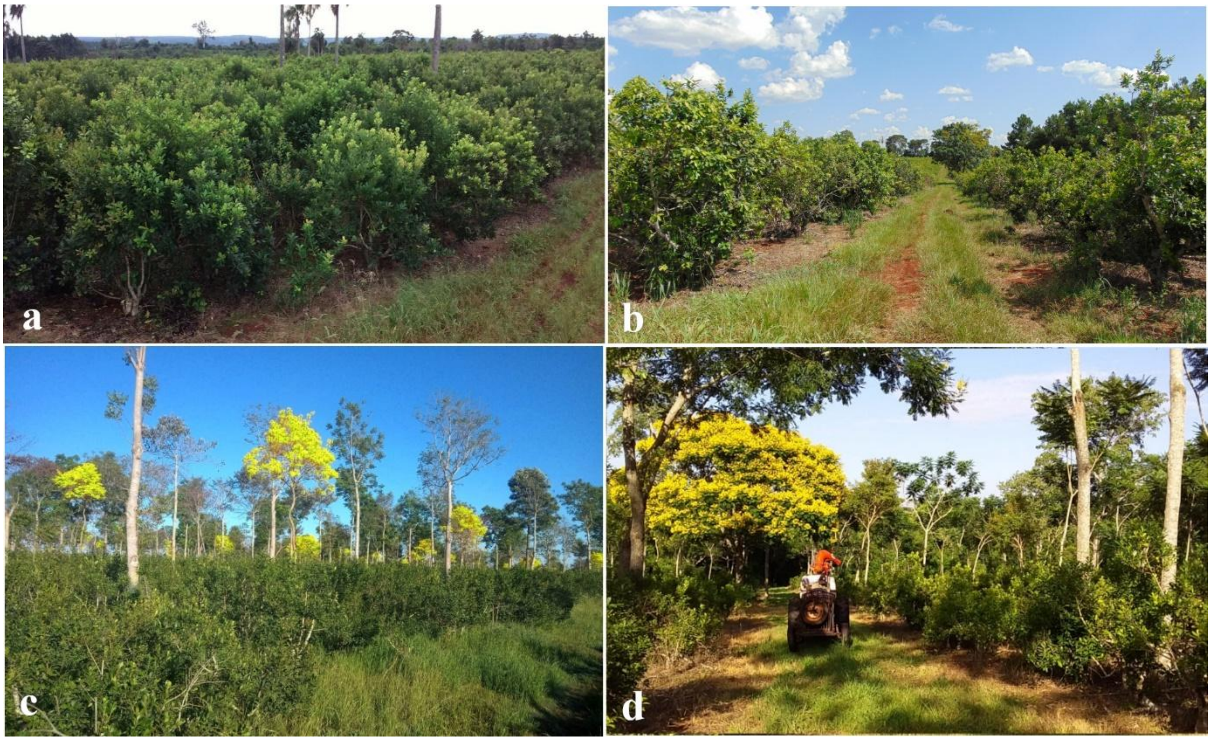

Supplementary Figure 2. a) A high-density plantation of yerba mate without trees; b) a traditional yerba mate plantation as an extensive monoculture; c) and d) high-density yerba mate plantations with trees. Photos taken in a) 2016, b) and c) 2018, d) 2019. The density is of approximately 3,700 mate plants and 246 tree plants per hectare, except in b) which is a conventional design typically with 2,200 plants per ha.

The images shown in Supplementary Figure 3 were taken in January 2022 at the peak of the local climate shock, in lots planted as high-density agroforestry lots of *I. paraguyensis* consociated with multiples species of trees. The images in Supplementary Figures 3 a)-c) correspond to the experimental trial lot of 10 has planted in 2010, while the images in Supplementary Figures 3 d)-f) correspond to a lot planted in 2015. In both cases it is immediately apparent that close association with trees, a), b), and d) protect yerba mate plants from the extremes of heat under drought conditions, compare with c) and e) and f), just a few meters away from trees. Supplementary Figure 4 shows images acquired in a lot in which yerba mate was planted preserving native trees that had recolonized an abandoned lot. The density of trees was incrementally increased to the standard in this work, approximately 740 trees per ha, which were subsequently thinned to approximately 246 per ha with managed regrowth to always have young trees growing, and tree branches and trunks decomposing in the ground. The distribution of tree ages and sizes is very heterogenous. This lot was the phenomenological basis for the systematic agroforestry layout discussed in the rest of this article. *I. paraguariensis* plants very close to trees stand the stress of extreme climate much better, panels b)-d), than plants without the immediate cover of trees, panel a).

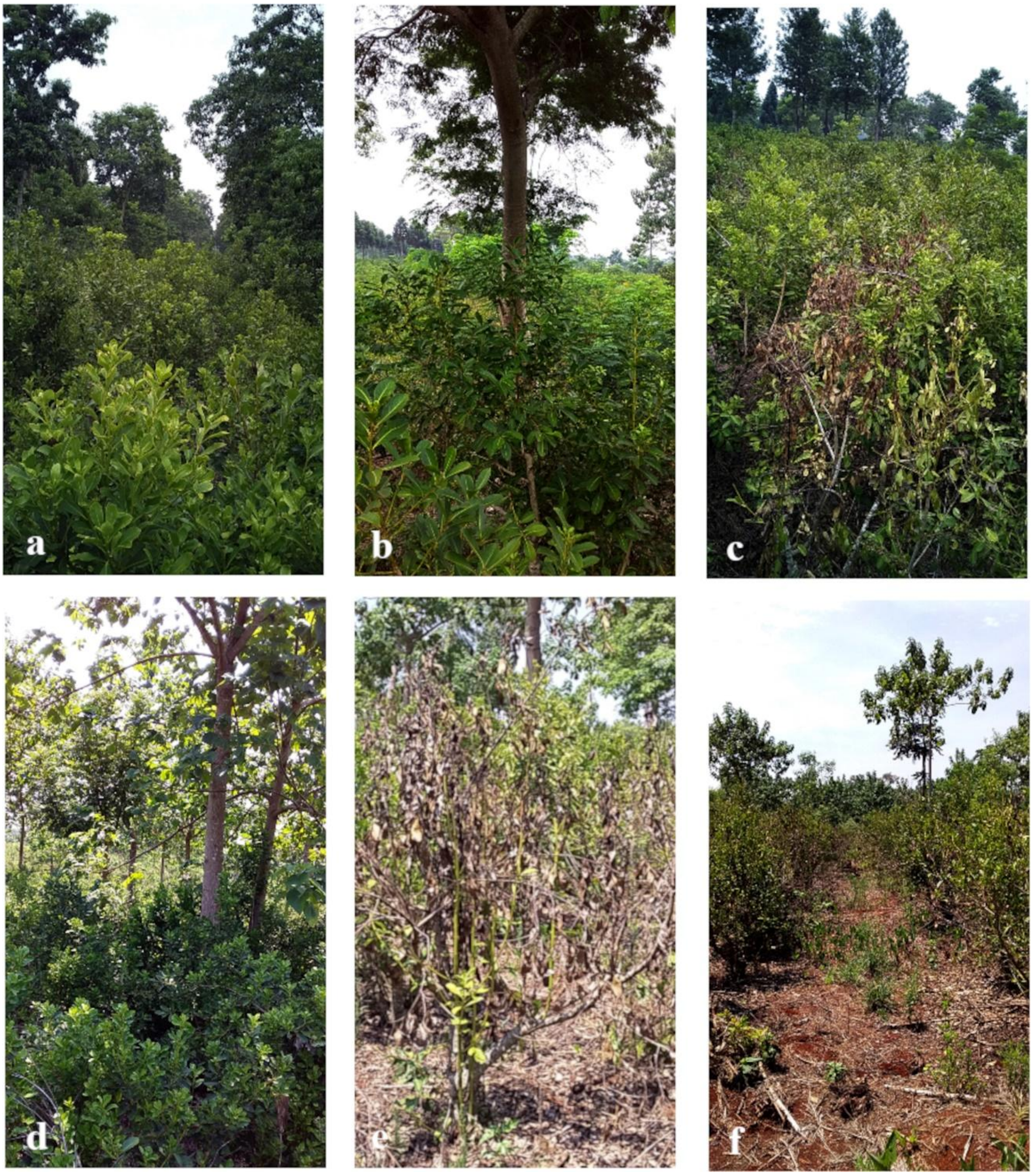

Supplementary Figure 3. Protection provided by trees against extremes of climate. Top row panels a), b), and c) show images from another sublot adjacent to the lots shown in Figures 1 a) and b), and Figure 3, spanning an area at the transition between intercalated trees, a) and b), to a no trees, c), less than 10 meters away. The absence of the microenvironment provided by the trees results in visible stress. The bottom row panels d), e), f) show images from a single lot, planted in 2015 with an heterogenous distribution of trees. While the area in d) has many trees and a microclimate, the areas in e) and f) a few meters away have no trees in high enough density to provide mitigation and contribute to a microclimate, and consequently show a high level of stress. The density is of approximately 3,500 mate plants and 740 trees per hectare.

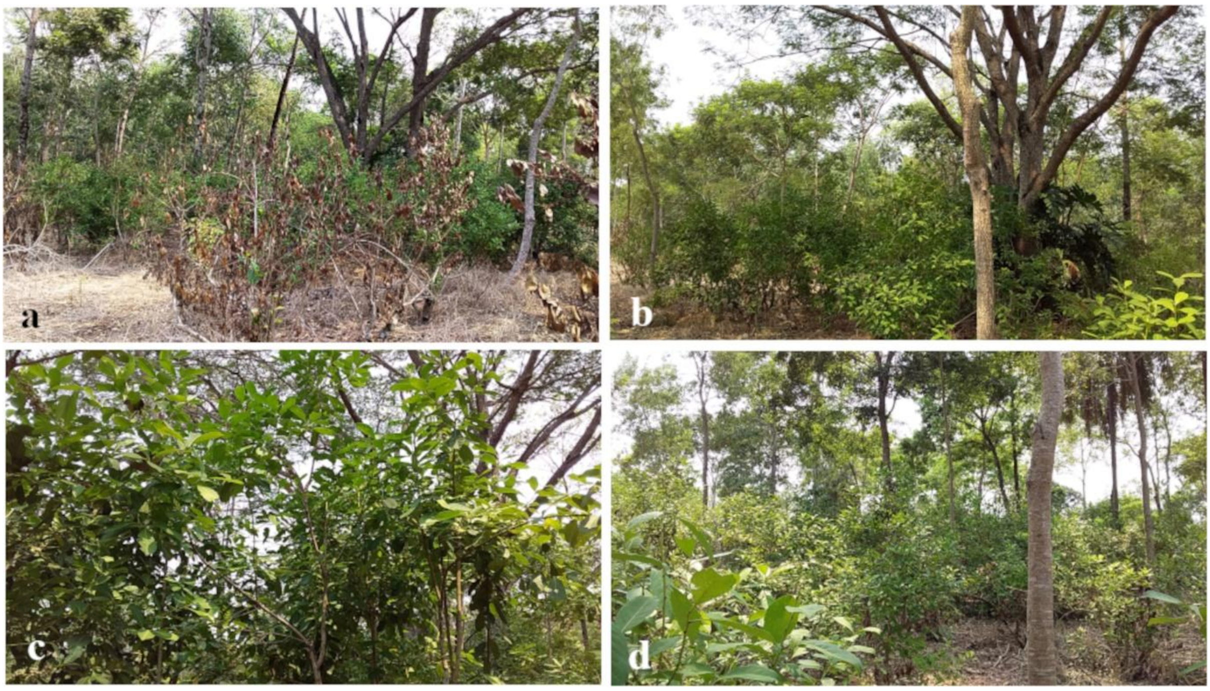

Supplementary Figure 4. Agroforestry through gradual enrichment. This is lot in which a high proportion of the trees pre-existed the yerba mate; new trees and mate were planted incrementally. In a) we see mate plants without tree cover drying up, and in progressive closeup views b) and c) we see mate plants with a healthy appearance closely associated with Cañafístola. Panel d) is a view of a different area of the same lot showing the heterogeneous distribution of trees of different ages and species with healthy mate plants. The density is of approximately 3,500 mate plants and 880 tree plants per hectare.

Pathways to Sustainable Agroforestry Development

To overcome the limitations of monoculture systems (Supplementary Figures 2a and 2b), we implemented and observed three primary approaches to establishing integrated agroforestry systems. The transformation of degraded soils into sustainable agroforestry systems can follow multiple routes, each with its unique process and benefits.

Artificial Forest Conversion: This method involves an initial phase of establishing an artificial forest on degraded land to facilitate soil restoration. After a period of soil improvement, *I. paraguariensis* is introduced within this established tree matrix. This approach was utilized in the *trial lot* described in Methods, underpinning all the data presented in this Results section and depicted in Figures 2 and 3, as well as Supplementary Figures 5, 6, and 7a.

Direct High-Density Multi-Species Planting: This route involves directly planting high-density stands of multiple tree species concurrently with yerba mate on previously cultivated or eroded land. While this method bypasses the initial soil restoration phase, it can require more external inputs during the establishment period. Supplementary Movies 1 and 2, and Supplementary Figure 7f, provide visual data on this approach.

Ecological Succession Enrichment: In abandoned agricultural lots undergoing natural ecological succession, we retained existing trees and augmented biodiversity by introducing additional tree species and *I. paraguariensis* to accelerate the development of a complex agroforestry system (Supplementary Figures 2d, 3d-3f, and 4).

Below is a summary of the three primary routes as practiced in your project.

Artificial Forest to Agroforestry Conversion:

- *Process*: Initially, create an artificial forest to initiate soil restoration.
- *Next Steps*: After a period, implant the agroforestry design within the rejuvenated soil.
- *Examples*: Demonstrated in Figures 2, 3, and Supplementary Figures 2 a), 3 a)-c), 5, 6, 7 a), b), and e).
- *Benefits*: This method allows for a gradual recovery of the soil's health, making it suitable for a diverse agroforestry system. The initial artificial forest phase helps in soil stabilization, nutrient cycling, and the establishment of a basic soil ecosystem structure.

Direct Conversion to High-Density Agroforestry:

- *Process*: Direct transformation of eroded, cultivated soil into high-density plantations, combined with multiple tree species.
- *Examples*: Illustrated in Supplementary Movies 1 and 2.
- *Benefits*: This approach is quicker, skipping the intermediate step of soil restoration through an artificial forest. It is suitable for soils that, despite being eroded, retain enough fertility to support the immediate introduction of diverse plant species.

Conversion of Abandoned Lots with Tree Conservation and Enrichment:

- *Process*: Transform abandoned agricultural lots, recolonized by native species, by conserving all present trees and enriching the tree density.
- *Examples*: Shown in Supplementary Figure 2 d) and Supplementary Figure 4.
- *Benefits*: This method leverages existing biodiversity and tree cover, enhancing it further for a balanced agroforestry system. It is particularly effective in areas where previous land use has left a variety of tree species that can be integrated into the new agroforestry design.

Evolution of the Integrated Agroforestry System: Over the course of this long-term project, our integrated agroforestry system has evolved to encompass nineteen tree species, including fourteen native or aboriginal and five exotic species (Table 1), integrated across over 200 hectares of *I. paraguariensis*. The initial selection of tree species (2003-2010) was guided by observational evidence suggesting beneficial consociation with *I. paraguariensis*, particularly with species such as *Caesalpinia paraguariensis*, *Araucaria angustifolia*, *Handroanthus impetiginosus*, and *Cedrela fissilis* (Supplementary Figure 2d, Supplementary Figure 4). This initial set was subsequently expanded to incorporate other species, including the legume *Anadenanthera macrocarpa* and *Toona ciliata*.

General Benefits Observed Across Approaches: Regardless of the specific establishment method, our integrated agroforestry systems have consistently demonstrated several key benefits. The incorporation of multiple tree species enhances the overall resilience and adaptability of the plantations, particularly in the face of environmental stresses. For example, during the extreme heat wave of 2022, *I. paraguariensis* plants grown in close proximity to various tree species exhibited significantly less stress compared to those planted in open monoculture conditions (as illustrated in Figures 2 and 4, and Supplementary Figures 3 and 4). This enhanced resilience and adaptability through tree consociation was a consistent pattern across all three establishment methods.

Comparative Analysis: The 10-hectare *trial lot*, established in 2010 with nine tree species (asterisked in Table 1) consociated with *I. paraguariensis* and a monoculture control (Methods, Supplementary Figure 1), provided quantitative data for comparing the performance of different agroforestry associations as detailed in the "Harvest yields" subsection. This controlled experimental design allows for annual measurements of various parameters, including yerba mate yield, insect populations, and soil composition, for each tree species 'treatment' and the control, providing a basis for direct comparisons. These data illustrate the variable impact of different establishment approaches on subsequent yields (Supplementary Figure 9). For example, the evolution of *I. paraguariensis* yields under different tree species canopy for the period 2014 to 2020 shows that the highest yielding consociation varied with the age of the plants. These observations are updated with the new climate regime impacting both tree management practices and the choice of species for different terrains. For the overall project, results are constantly evolving.

Documentation of Everyday Life and Biodiversity in this Agroforestry System

In Supplementary Figures 5 to 7 we document a range of attributes of this agroforestry system encountered in everyday life outside the extremes of climate. They also document the impact on workers' daily tasks and work environment, providing a better microclimate throughout the seasons and enriching the diversity of know-how and expertise they acquire. These attributes become critically important during shocks of extreme climate, as they provide mitigation and help to adapt. There is a synergy among all these aspects which underpin a considerably higher resilience for the activity.

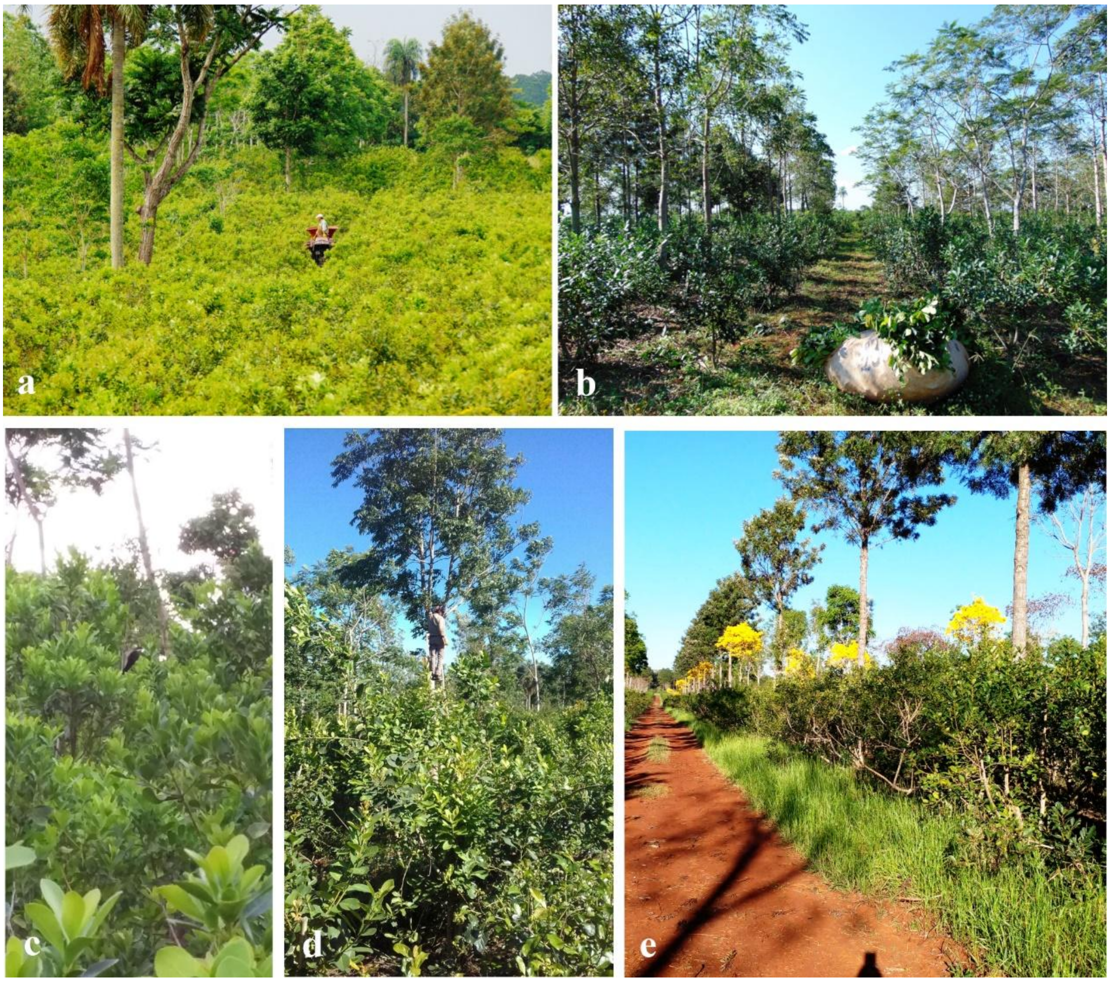

Supplementary Figure 5. Key aspects of the agroforestry system before the climate shock. Panel a) shows a control lot without trees left within the forested standard practice. Before the extreme weather events *I. paraguariensis* plants fared well with or without trees. The tractor is distributing grounded dolomite, a Ca-rich stone. Panel b) shows another view of the same 10 has lot just as harvest was being wrapped-up. A bundle of leaves is on the ground, which is covered with docile herbs (not Gramineae or grasses). The harvest is measured for each sub-lot with a single tree species to compare the results of consociation and the control lot without trees. It is normal to find birds, panel c). Part of the tree branches are trimmed, panel d), and left to rot on the ground, which is also covered with ryegrass and rye during the summer months e). Photos taken in summer of 2018, a), winter of 2017, b), spring of 2020, d), and spring of 2021, c) and e). The density is of approximately 3,700 mate plants and 880 tree plants per hectare.

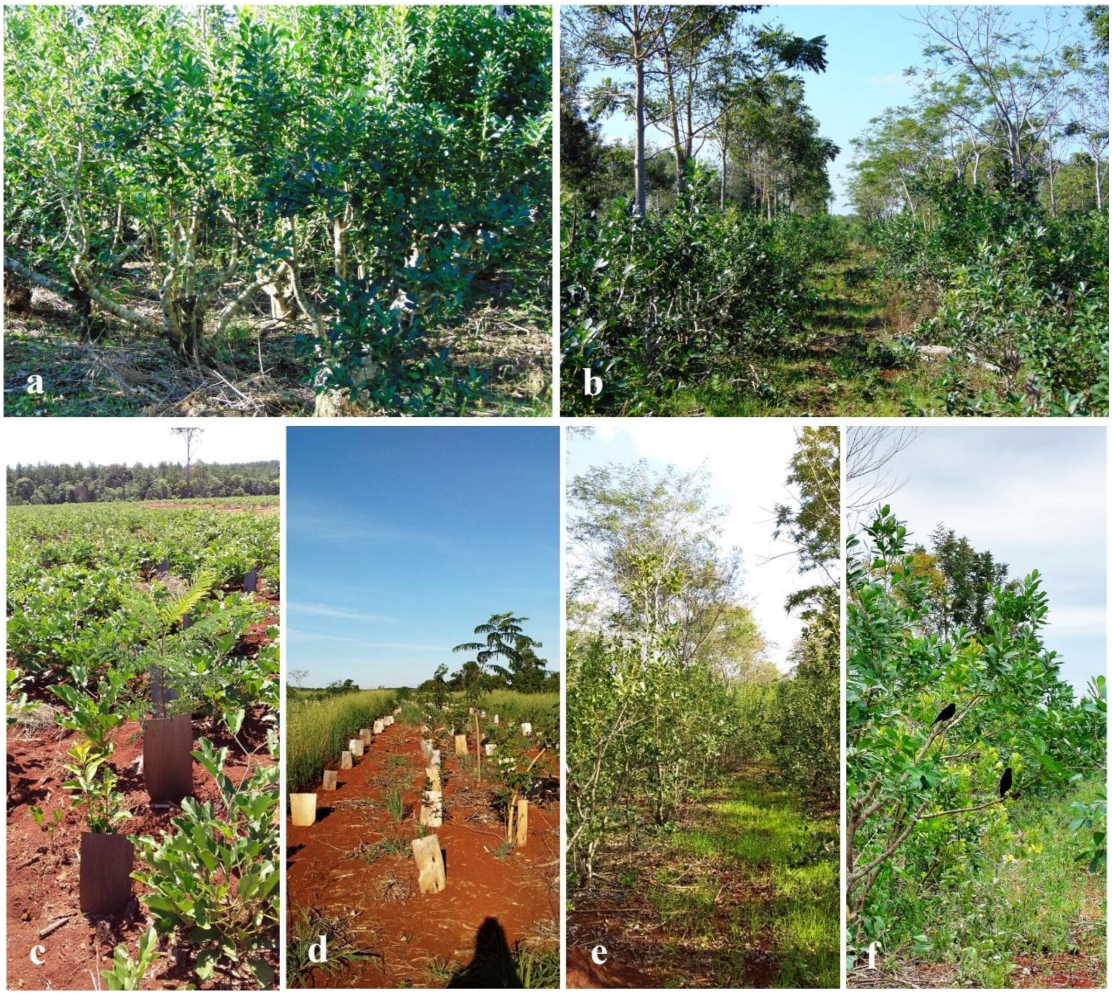

Supplementary Figure 6. Management of ground cover. Panels a) and b) are images of established high-density mate plantations showing the desired ground cover. There aren't highly detrimental Gramineae or grasses, which the plant density compete out with the help of labor during the summer months. Tree trunks and branches are left to rot on the ground increasing the content of organic matter. During the implantation of the ground is covered with legumes in summer, c), and ryegrass during winter, d); the grasses are manually extracted resulting the aspect shown in d); as the plants grow the ground remains covered by the coverage given by mate plants and trees with high density, and planted green covers, e), which grow less due to the diminished light they receive. Birds find plenty of branches to support foraging of insects, f); the ground is covered by planted green cover as before, plus native docile herbs. Birds incorporate the system as part of their habitat.

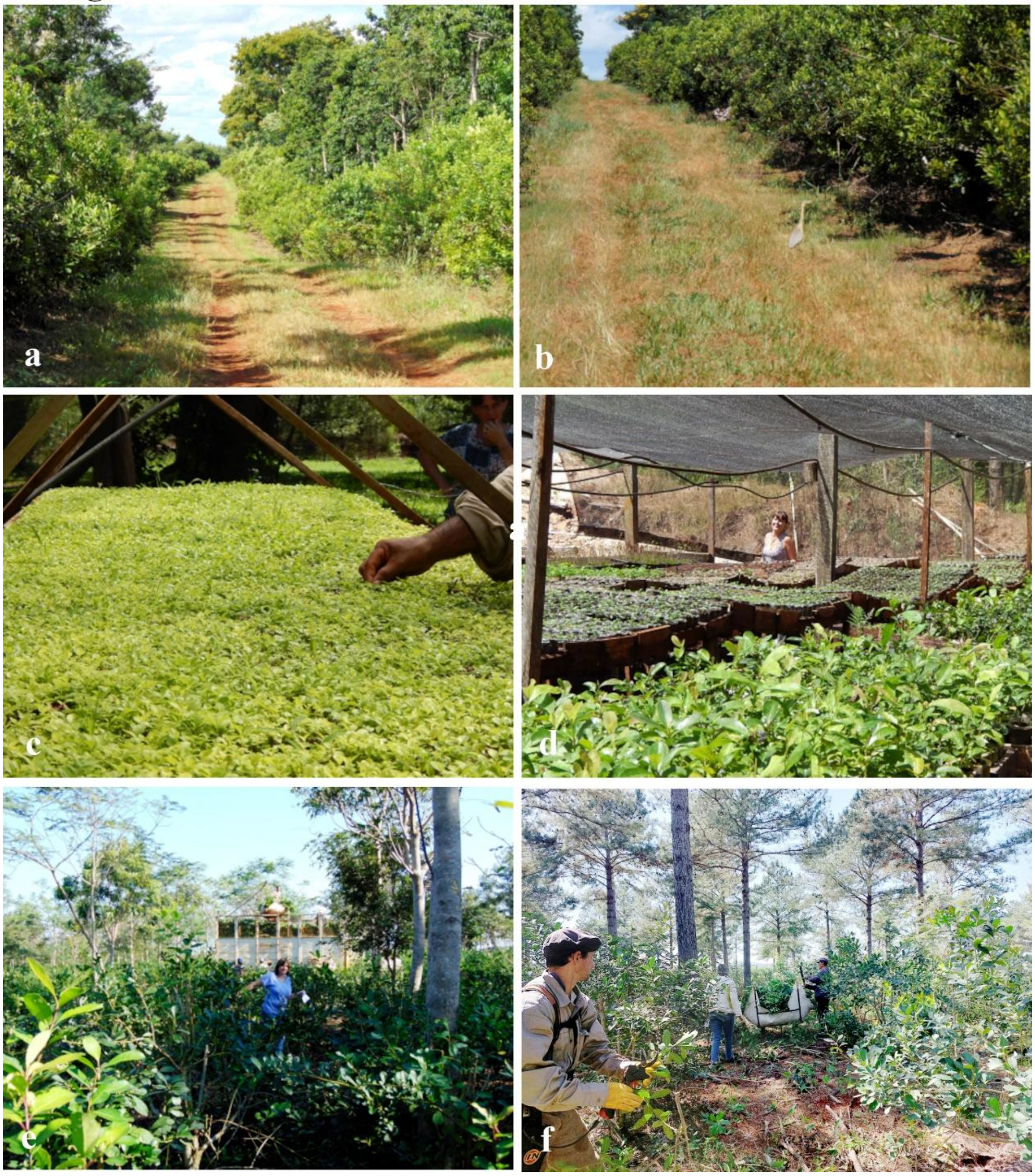

Supplementary Figure 7. Microclimate and work environment. Panels a) and b) are images taken in January of 2022, at the peak of the climate extreme showing a fully green context and wildlife. Panel c) shows tree seedlings being transplanted to small pots for further growth, panel d); photos acquired in 2017. Panels e), photo 2017, and f), photo 2022, show a range of tasks during the crop cycle including cutting and trimming, weighting, and annotating measurements for bookkeeping and statistics.

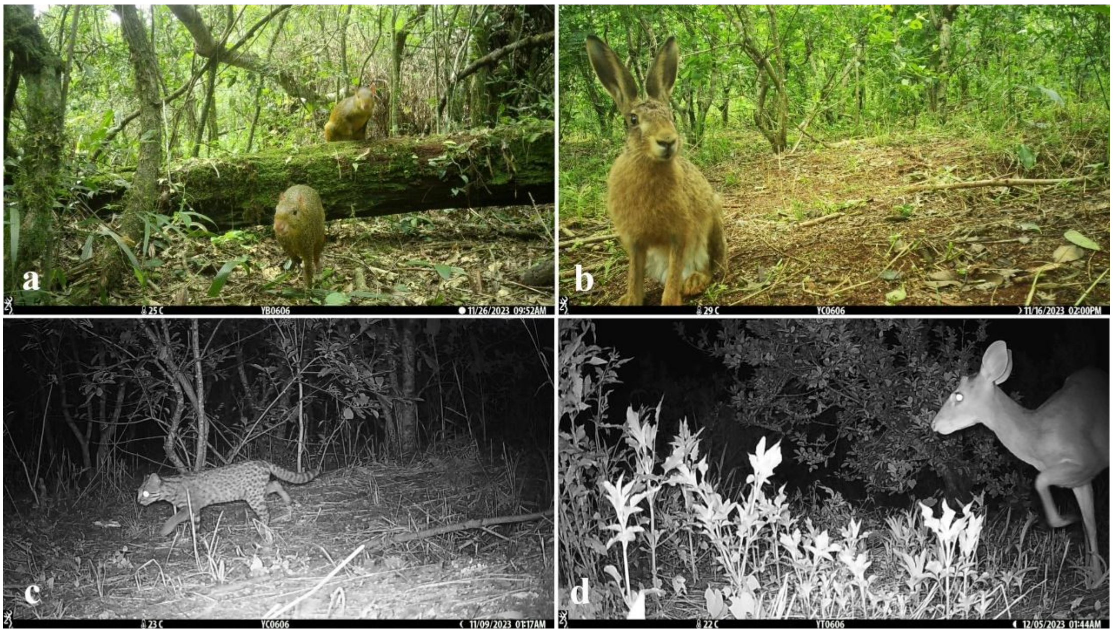

Supplementary Figure 8. Numerous small mammals native to this region have returned, making this agroforestry system part of their natural habitat. Panel a), "agutí bayo" (*Dasyprocta azarae)*, a native forests rodent. Panel b), liebre común o liebre europea (*Lepus europaeus*). Panel c), tirica o (*Leopardus guttulus*), a small wild feline. Panel d), corzuela colorada o *guasú-pihtá* (*Mazama rufa*) un animal con morfotipo de cérvido. Image by Paula Cruz and Victoria Gross, see Supplementary Discussion, Experimental Context below.

Supplementary Movie 1. Overview of an agroforestry lot implemented on a previously cultivated, and degraded lot without a fallowing cycle. The lot is 5 years of age, the movie was taken from a cell phone during spring.

Supplementary Movie 2. Overview of the same lot as in the previous movie, taken during winter immediately after the cropping cycle a year after Supplementary Movie1.

The documentation in these figures underscores the multifaceted benefits of the agroforestry system. A comprehensive approach improves the overall quality of the environment and the livelihoods of those who work within it. The increased resilience to climate extremes, coupled with the enhanced work environment, demonstrates the system's effectiveness in providing sustainable solutions to contemporary agricultural challenges, including the following key aspects:

Microclimate Regulation

- The agroforestry system creates a stable microclimate, which is beneficial throughout the year.
- It offers cooler conditions in summer and warmer environments during winter, making it a comfortable workspace for the workers.

Impact on Workers' Tasks and Skills

- The diverse environment enriches the variety of tasks and skills required by the workers.
- Workers gain knowledge and expertise in managing a range of plant species and agricultural practices unique to agroforestry.

Resilience to Extreme Climate Events

- The system demonstrates increased resilience during extreme climate events.
- The diversified plant cover and soil management practices help mitigate the impacts of extreme weather, such as intense rainfall, droughts, and heatwaves.

Synergy Among Various Aspects

- There is a notable synergy between the ecological benefits, restoration of biodiversity, and the improvement in work conditions.
- The system not only enhances biodiversity and soil health but also provides a safer and more comfortable work environment.

Documentation and Observation

- Supplementary Figures 5 to 8 serve as a visual record of the agroforestry system's attributes.
- They provide evidence of the system's effectiveness in everyday scenarios, outside of extreme climate conditions.

Supplementary Discussion

Measurements in context

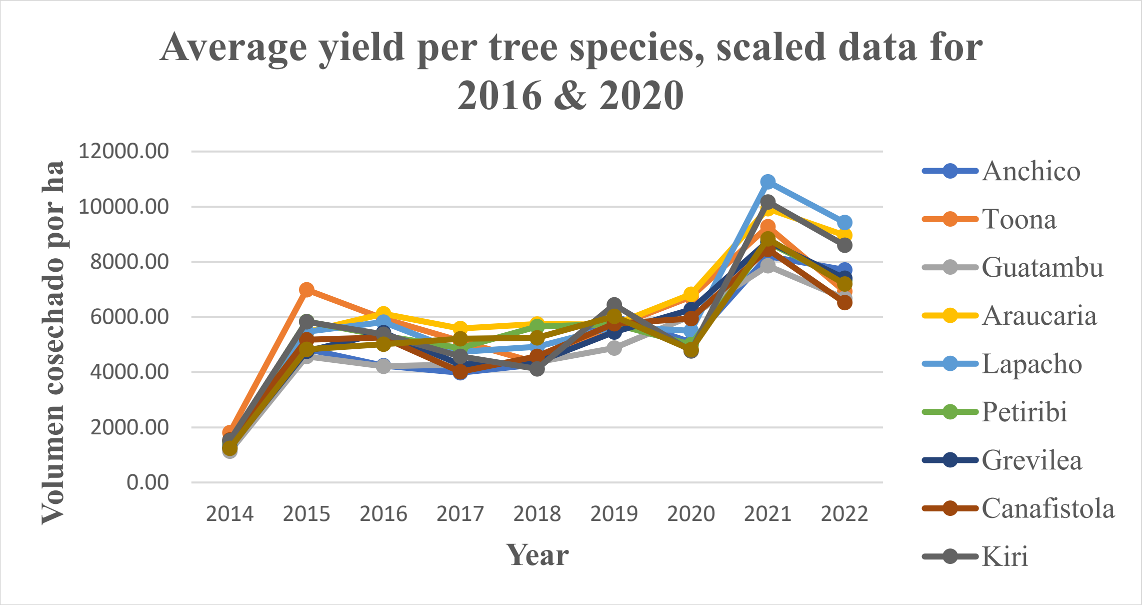

Supplementary Figure 9. The evolution of yerba mate yields under different tree species canopy for the period 2014 to 2020. Yerba mate is planted in 5 lines or rows with trees in the central row. In 2016 and 2020 only the central row was measured, in 2016 due to heavy rains and in 2020 due to Covid-19 which made labour considerably more difficult.

Supplementary Table 1. Comparison of soil tests results for *I. paraguariensis* (yerba mate) monocultures, from neighbouring lots, with the agroforestry system. Values with fonts in blue in Supplementary Table 1A are examples of important improvement achieved with agroforestry relative to monoculture while values with fonts in read, in Table 1B, are examples of detrimental values.

The organic matter content is higher in the agroforestry trial lot than the reference monoculture lots. As these soil tests represent the initial stages of a perennial agroforestry system, much progress remains to be achieved. Of great impact, the acidity in agroforestry lots is lower, with higher “base saturation” levels, a measure of the capacity to exchange cations. Additionally, the level of aluminium is much higher in the reference monoculture lots than in the agroforestry lots, at levels that are toxic for this activity. The level of extractable Phosphorous contains the routine applications of standard commercial triple nitrogen (N), phosphorus (P) and potassium (K) (NPK) fertilizers at the rate of 50 kg per 1,000 kg of harvested yerba mate distributed uniformly across whole lots, but the level of available phosphorous improves in the agroforestry lots as expected with higher soil organic matter content and lower acidity. This gradual, hard-won improvement just shows how critical it is to prevent soil degradation in the first place.

Supplementary Table 1A. Soil elemental composition for restored soil and agroforestry trial lot.

**Field Identification**	**Lot 6**	**Agroforestry Trial Lot**	**Lot 7**
**Agricultural activity**	Restored, high-density y. mate	Restored, y. mate agroforestry	Restored, y. mate agroforestry
**Sampling Depth (cm)**	20	20	20
**Oxydazable Organic Matter (OOM%)**	2,08	1,99	2,38
**Total Organic Matter (TOM%)**	2,71	2,59	3,10
**Carbon (Readily Oxidazable -ROC %)**	1,21	1.,16	1,38
**Total Nitrógen (%)**	0,17	0,18	0,20
**Ratio C/N**	9,15	8,66	8,95
**Extractable Phosphorous P_2O_5 (ppm)**	13,91	10,54	9,58
**Exchangeable Potassium K (meq/100g)**	0,21	0,26	0,26
**Exchangeable Calcium Ca (meq/100g)**	2,59	5,63	5,90
**Exchangeable Magnesium Mg (meq/100g)**	1,14	1,02	0,78
**Exchangeable Sodium Na (meq/100g)**	0,04	0,04	0,05
**Sum Bases (meq/100g)**	3,99	6,95	7,00
**Exchangeable Acidity (meq/100g)**	13,30	1,.33	11,72
**Cation Exch. Cap. CEC Total (meq/100g)**	17,29	1,.27	18,71
**Base Saturation V (%)**	23,05	40,25	37,38
**pH Water (1:2.5)**	5,00	5,26	5,35
**pH ClK (1:2.5)**	3,88	4,20	4,15
**Exchangeable Aluminium Al (meq/100g)**	0,85	0,32	0,02

Supplementary Table 1B. Soil elemental composition for monoculture lots without a restoration cycle.

**Field Identification**	**Soil 9A S. Pipo 1**	**Soil 9A S. Pipo 2**	**Soil 9A S. Pipo 3**
**Agricultural activity**	Monoculture y. mate since 1930s	Monoculture y. mate since 1930s	Monoculture y. mate since 1930s
**Sampling Depth (cm)**	20	20	20
**Oxydazable Organic Matter (OOM%)**	1,26	2,23	2,21
**Total Organic Matter (TOM%)**	1,64	2,91	2,88
**Carbon (Readily Oxidazable -ROC %)**	0,73	1,30	1,28
**Total Nitrógen (%)**	0,11	0,14	0,15
**Ratio C/N**	8,48	12,39	11,02
**Extractable Phosphorous P_2O_5 (ppm)**	4,76	7,82	4,04
**Exchangeable Potassium K (meq/100g)**	0,07	0,10	0,09
**Exchangeable Calcium Ca (meq/100g)**	1,51	1,81	1,82
**Exchangeable Magnesium Mg (meq/100g)**	0,96	1,15	0,71
**Exchangeable Sodium Na (meq/100g)**	0,04	0,01	0,04
**Sum Bases (meq/100g)**	2,58	3,07	2,66
**Exchangeable Acidity (meq/100g)**	13,72	12,47	13,27
**Cation Exch. Cap. CEC Total (meq/100g)**	16,30	15,54	15,93
**Base Saturation V (%)**	15,85	19,74	16,72
**pH Water (1:2.5)**	4,66	4,89	4,64
**pH ClK (1:2.5)**	3,78	3,96	3,86
**Exchangeable Aluminium Al (meq/100g)**	2,80	1,56	1,60

Experimental Context

This project spans over 200 ha located in three blocks at approximately 15 km from each other. There is abundant granular, fine-grained context that does not belong in the main manuscript. This section expands the field-work narrative of the experimental design with comprehensive contextual information for the procedures employed, the geographical locations of data sources, and relevant visual information to support the methodological framework.

The region is defined by its humid subtropical climate without dry season, with the areas involved in this work situated at approximately 175 m to 220 m above mean sea level. From year 1967 to year 2020 the average annual rainfall was 1998 mm, with a minimum of 1120 (2004) mm and a maximum of 3034 (2014) mm. Towards the end of 2020 and during 2021 and 2022 the weather phenomenon called "El Niño" brought extremes of heat and drought, while during 2023 "La Niña" brought extremes of rainfall. The total rainfall for 2020, 2021, 2022, and 2023 was 1310 mm, 1536 mm, 2136 mm, and 1900 mm respectively. Typically, temperatures range from a minimum just above zero degrees Celsius to approximately 36 degrees Celsius. The minimum historical temperature was recorded on July 18, 2017, with -8 degrees Celsius at 5 cm above the soil, and the maximum historical temperature on January 24, 2022, was of 42.5 degrees Celsius[9]. We provide below Extended Background and Detailed Methodological Context for the data presented, and coordinates with cadastre identification for all the lots from which the data presented here were obtained. In addition, and beyond the images presented in the figures and the data presented in tables, data used on a routine basis includes soil sampling and chemical analysis, insect traps count as monitoring exercises, photos of soil temperature measurements, humidity measurements, actual data on water quantity and frequency necessary under different conditions for watering seedlings during droughts.

Main Manuscript Table 1. Tree species used in the multi-species agroforestry system presented here. The density is of approximately 3,700 mate plants and 246 trees per has during 2015-2018, 123 trees and 123 pruned regrowing trees since 2019. These nineteen (19) species of trees have been planted within yerba mate lots since the early 1990s over a total area of approximately 200 has. New species are planned starting 2022. Toona, Grevilea, and Kiri, are exogenous species with synergy with yerba mate cultivation from anecdotal evidence according to the perceived vitality and health of consociated yerba mate. Two lots totalling 10 has were used as an experimental pilot program designed for measurements. The pilot was implanted with nine (9) species, Toona, Grevilea, Kiri, Lapacho, Guatambu, Petiribi, Anchico, Araucaria, and Cañafístola, following a plan with identical layout for all species in four (4) sublots and four (4) control sublots without trees. This pilot or test lot is identified as Lots 1 and 3 within "Lote XII" (~98 has), cadastre of the municipality of Santo Pipo, Misiones, Argentina (https://earth.google.com/ 27° 8'34.77"S, 55°23'37.18"W). This layout results in approximately 1 ha per tree species and control area without trees. The "agroforest trial plots" are used to measure yerba mate production volumes for each consociated forest species and non-forested plots each year, acquire data on insect counts, soil analysis, and during extreme heat and drought to measure temperatures and verify soil moisture compared to traditional non-forested cultivation.

Main Manuscript Table 2. Comparative yields for agroforestry and monoculture. Accumulated crop yield per tree species and test lots without trees for the period 2014-2022 for the agroforestry trial lot. As shown in Supplementary Figure 1, this experimental trial lot spans two adjacent lots with a combined area of 10 has, divided in 4 blocks with 10 identical sublots following a plan with identical layout for all species and controls, one for each species of tree and one control without trees. The same number of yerba mate plants and trees of each species were planted in each lot, except the control which has no trees. Each species and control cover 1 ha, with approximately 3,700 yerba mate

plants and 246 trees. Of these trees, since 2019, half are managed as regrowth after thinning an adult tree every 2 trees, sequentially, and the other half are managed as adult trees, 123 trees per ha. The same dose of fertilizer is applied to the whole trial lot, the reposition dose for yerba mate which for N-P-K-Ca-Mg mixes is 9-1-8-2-2 kg per 1 kg of mate harvest. We consider the 10 has average for the dosage of fertilizers.

The harvest of yerba mate is done manually, with each person first gathering the harvest in packages of approximately 100 kilos, as in Supplementary Figure 5 b). The weight of each package is weighed with a portable scale (a pylon balance or an electronic pylon balance - Scale Hook with 0.1 kg measurement uncertainty) with an error of less than approximately 1% when considering all the handling including a fistful of leaves of a few hundred grams randomly dropped or added, and collected in specially prepared truck chassis, then transported to the roaster and dryer facility. Note that 10,000 kg result from 100 measurements of approximately 100 kgs with errors normally distributed, so the propagated uncertainty is $\sqrt{N}$ x1 kg = $\sqrt{100}$ x1 kg $= 10$ kgs. The total volume for the period 2014-2022 accumulated per species is approximately in the range 45 K kgs to 52 K kgs, i.e. ~ 50 K kgs, from ~ 500 weight measurements. We are not interested in the mean, but the total volume obtained per tree species. The appropriate error propagation is the propagation of the uncertainty in a weight measurement. The yield of mate can be compared directly among species and with the control. The nine (9) species of trees are Toona, Grevilea, Kiri, Lapacho, Guatambu, Petiribi, Anchico, Araucaria, and Cañafístola. The trial lot spans lots 1 and 3 within "Lote XII" (~98 has), cadastre of the municipality of Santo Pipo, Misiones, Argentina (Supplementary Figure 1). All the differences in accumulated yield are several times the propagated error.

Main Manuscript Table 3. Counts of insects in traps. Number of "psilido" (*Gyropsylla spegazziniana***)** individuals counted in Moericke traps within the agroforestry trial lots and in standard monoculture lots without trees; images and text box below provide details. Identification of sampling locations: The insect counts labelled as "counts with trees" were done in the agroforestry pilot or test lot identified as Lots 1 and 3, within "Lote XII", cadastre of the municipality of Santo Pipo, Misiones, Argentina. The insect counts labelled as "counts without trees" were done in Lot 12, within "Lote XII" above, without trees at the time (https://earth.google.com/ 27° 8'26"S, 55°23'55"W).

Supplementary Figure 10. Moericke traps.

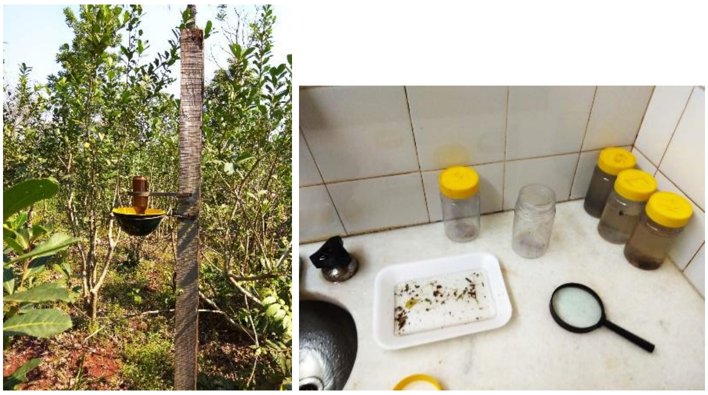

Left: Insect traps are "home-made", using commercial Tupperware as a box, with water, and an inverted beer bottle to replenish the water as it evaporates (Moericke traps). Right: Counting of insects in the laboratory; each bottle corresponds to a trap. Estimated errors are below 10%.

Main Manuscript Table 4. Averaged selected results or soil tests for the trial lot. Samples were collected from each location using a 0.25 m ring sampler at 0.2 m of depth. Each sample is a composite of six subsamples. All soil tests analysis, including Tables 4, Supplementary Tables 1A and 1B, were carried out at the Instituto Nacional de Tecnología Agropecuaria (INTA), Estación Experimental Agropecuaria Cerro Azul (EEA Cerro Azul), Ruta Nacional 14. Km. 836, Cerro Azul, Misiones C. P. (3313). As reported in the main manuscript Methods section, the method used for

estimations of soil organic carbon (ROC %) is based by the chromic acid titration method by Walkley and Black*, standardized in Argentina by the Instituto Argentino de Normalización y Certificación (IRAM) with norm "IRAM-SAGyP 29571-3:2016 Parte 3: Determinación de Carbono orgánico oxidable por mezcla oxidante fuerte- microescala". Oxydazable Organic Matter (OOM) and Total Organic Matter (TOM) are obtained from ROC by multiplicative factors that depend on the context. (*) "Walkley, A.J. and Black, I.A. (1934). Estimation of soil organic carbon by the chromic acid titration method. Soil Sci. 37, 29-38". The values reported were computed from 156 individual samples. Soil tests for each individual species have been averaged in aggregate; the standard deviations are in all cases below 12 %, Table 4 as well as Supplementary Table 1. There are species-specific results differences which are part of work in progress (A. von W., N. M. B.) and will be published in a more specialized article.

Supplementary Table 1.

A. Trial lot, Lots 1 and 3, are within "Lote XII" (~98 has), cadastre of the municipality of Santo Pipo, Misiones, Argentina, (Google Earth, https://earth.google.com/ 27° 8'30.47"S, 55°23'32.72"W and 27° 8'34.77"S, 55°23'37.18"W respectively). Soil sampling and analysis reported in this table were done in September 2019, with laboratory identification numbers 6645 lot 7, 6646 lot 6, 6650 lot 3, and 6652 lot 1. The values from lots 1 and 3 have been averaged. Estación Experimental Agropecuaria Cerro Azul INTA (EEA Cerro Azul), Ruta Nacional 14. Km. 836, Cerro Azul, Misiones C. P. (3313), Argentina, see below.

B. Soil chemical analysis, traditional monocultures. Soil 9A S. Pipo 1, is "Lote 14" S. Pipo 2 is "Lote 13"**;** S. Pipo 3 is "Lote 5"; cadastre of the municipality of Santo Pipo, Misiones, Argentina (https://earth.google.com/ 27° 7'49.05"S, 55°23'15.27"W; 27° 8'16.42"S, 55°23'39.69"W; 27° 7'50"S, 55°25'12"W respectively). Soil sampling and analysis reported in this table were done in September 2019, with laboratory identification numbers 6641 S. Pipo 1, 6625 S. Pipo 2, 6331 S. Pipo 3, Estación Experimental Agropecuaria Cerro Azul INTA (EEA Cerro Azul), Ruta Nacional 14. Km. 836, Cerro Azul, Misiones C. P. (3313), Argentina.

Supplementary Figure 8. Images documenting small mammals were acquired by Paula Cruz and Victoria Gross as part of a Project titled "Relevamiento de mamíferos en plantaciones de yerba mate (*Ilex paraguariensis*) de Misiones" ("Survey of mammals in yerba mate plantations in Misiones"), taking place in "Lote XII" (~98 has), cadastre of the municipality of Santo Pipo, Misiones, Argentina (https://earth.google.com/ 27° 8'34.77"S, 55°23'37.18"W). The camera trap (Browning BTC-5DCL) was located off-road and attached to the base of a yerba mate plant, at a height of about 30 cm above ground level. The camera was set to take three successive pictures per trigger with no delay between detections. It was active for 36 days from November 08 to December 12 of 2023 and took 323 photographs. This project is led and carried out by the researcher Paula Cruz from the Institute of Subtropical Biology (IBS, Iguazú node – National University of Misiones (UnaM)- Consejo Nacional de Investigaciones Científicas y Técnicas -CONICET (National Scientific and Technical Research Council), and Victoria Gross, student of the Park Ranger career, from the Faculty of Forest Sciences, of the National University of Misiones (UnaM)."

Geographical location of the lots of cultivated land shown in the images.

Main Manuscript Figure 2. Panel a) is an image of lot 7 (~10 has) and panels b), c), and d) show images from lot 3 within "Lote XII", cadastre of the municipality of Santo Pipo, Misiones, Argentina (https://earth.google.com/ 27° 8'40.35"S, 55°23'50.97"W; 27° 8'34.77"S, 55°23'37.18"W). The yerba mate and trees were planted in 2011 and 20 12 for a) and 2010 for b)-d). "Lote XII" will refer to this

registry from here onward. All lots shown from this cadastre location, Lote XII, share the same history. They were first planted with yerba mate in 1934 on land cleared from native forest and cultivated without interruption until 1992. Between 1992 and 1996 artificial pine (Pinus elliottis) forests were planted on the exhausted yerba mate lots, and from 2010 onward they were converted to the current agroforestry system through the simultaneous implantation of multiple varieties of trees species and yerba mate. Photographs taken in January 2022.

Main Manuscript Figure 3. Panels a) and b) show images from lot 7 within "Lote XII" as in Figure 2 a) and b) but photos taken in 2018 when the plantation was 7 years old. Panel c) is from "Lote 37d" cadastre of General Urquiza, Misiones, Argentina as in Supplementary Figure 4 (https://earth.google.com/ 27° 4'39.51"S, 55°26'34.02"W). This lot was planted in 2005 on previously abandoned agricultural land. Native tree species that had recolonized the lot were left in place and the lot was enriched following Table 1; photos taken in 2017. Panel d) is from experimental pilot lot 3 within "Lote XII", as in Figure 3; photos taken in 2017 when the plantation was 7 years old.

Supplementary Figure 2. Panel a) shows a high-density plantation without trees, and b) a typical large-scale monoculture of yerba mate. Panels c) and d) show cultivation as a multi-species agroforestry design, c) planted in 2015 on degraded land cultivated since 1934 with yerba mate, and d) planted in 2005 on an abandoned orange orchard with many native tree species reintroduced naturally. Panels a) and c) are images of yerba mate planted within "Lote XII", cadastre of Santo Pipo, Misiones, Argentina (Lots 6 and 8 respectively; https://earth.google.com/ 27°8'32"S, 55°23'49"W; 27°8'42"S, 55°24'W). Photographs taken in January 2022. Panel b) "Lote 68a ((https://earth.google.com/ 27° 5'10.23"S, 55°25'33.25"W), and "d) "Lote 37d", cadastre of General Urquiza, Misiones Argentina (https://earth.google.com/ 27° 4'39.51"S, 55°26'34.02"W), photograph taken in 2018.

Supplementary Figure 3. Protection against extreme heat provided by the microclimate afforded by tress. Panels a), b), and c) show images acquired in the agroforestry experimental pilot lot, lot 3 within "Lote XII", as described in Figure 3 main text and above in S. M. Data. Panels d), e), and f) show images taken in "Lote 1b", cadastre of the municipality of General Urquiza, Misiones, Argentina (https://earth.google.com/ 27° 2'9.78"S, 55°25'32.34"W). This lot was planted in 2015 on land cleared of forest with a few standing trees left over, subsequently enriched with several species of trees. Mate plants in the areas cleared of trees, f), show a striking level stress relative to plants near trees, d) and e). The microclimate and interactions provided by trees provides significant higher well-being. Photographs taken in January 2022.

Supplementary Figure 4. Incremental agroforestry. All images are taken from "Lote 37d" of the cadastre of the municipality of General Urquiza, Misiones, Argentina (https://earth.google.com/ 27° 4'39.51"S, 55°26'34.02"W). This lot was planted in 2005 on an area abandoned for grazing in the decades following WWII, in which many native trees had recolonized the lot, reintroduced by natural processes. The recolonized native trees were enriched to reach the density used in this agroforestry system. Photographs taken in January 2022.

Supplementary Figure 5. Panels a) and b) show images of the 10 has pilot agroforestry experimental lot used for systematic data acquisition, lots 1 and 3 within "Lote XII", cadastre of the municipality of Santo Pipo, Misiones, Argentina. Photos taken in 2018. a) is a view of one of the control sublots without trees. The tractor has just gone from the forested area into one of the control sublots without trees within lot 3. Four such control lots of approximately ½ has each were designed within the two lots spanning 10 has, therefore allowing for systematic comparisons. b) is a view of the same lot from the side with trees taken during the winter crop in 2018. Leaves are typically wrapped in packages of

approximately 90 kgs to 100 kgs, such the one in the image. The lot had been trimmed to collect the leaves, which is the crop of mate tea. The plants of yerba mate, in this design, are left with considerable foliage, as shown. Panels c) and e) are images from lot 8 and lot 7 within “Lote XII” acquired in 2021, and panel d) is an image from lot 2 within “Lote XII”, acquired in 2020. The presence of trees supports birds, c), which control insects even if the trees are trimmed, d), to permit more sunlight for the development of the plants of *I. paraguariensis* and the green covers, as well as valuable logs, e).

Supplementary Figure 6. a) Lot 6 within “Lote XII” and b) lot 1 within “Lote XII”, photos acquired in 2017. Panels c)-f) images from “Lote 68” of the cadastre of the municipality of General Urquiza, Misiones, Argentina (https://earth.google.com/ 27° 5'10.23"S, 55°25'33.25"W); c)-e) photos acquired in 2020, and f) in 2022.

Supplementary Figure 7. Panels a) and b) show images acquired in January 2022, during the peak of the extreme climate period, within “Lote XII”. Panels c) and d) are images of the trees and *I. paraguariensis* nursery, in “Lote 6A, municipality of Santo Pipo, Misiones, Argentina (https://earth.google.com/27° 7'45"S, 55°25'07"W). The photos were acquired in 2017. Panel e), photo in lot 7 within “Lote XII”. Panel f), photo acquired in Lote 84C, municipality of Bernardo de Irigoyen, Misiones, Argentina, in September of 2022 (https://earth.google.com/ 27° 6'2"S, 55°21'12"W).

General considerations regarding data modalities and their range of maximum validity. “Image Data” provides context that allows numerical data to be interpreted with more perspective. Numerical data is limited and expensive; even with all the numerical data in real time it would be impossible to make projections without the information from the images. The image data shows the development and size of each forest species; crown type and comparative growth between species; state of the yerba mate according to color and abundance of leaves (with the Kiri the plants have much more foliage but due to excess shade the production in kg is lower); appearance of the bark of the yerba mate; sun damage; soil condition, compaction and appearance of weeds, types of weeds; resolution of excess water during heavy rainfall; hospitability of the environment during periods of excessive heat or unusual frost. The numerical and image data "inform" each other and allow a better understanding of the whole (of the system).

Numerical data: harvest volume of yerba mate by consociated forest species, that is, production volume in Kg for each forest species with which yerba mate is linked in specific plots; soil analysis; survey of harmful insects using traps and manual monitoring, e.g., psyllid (*Gyropsylla spegazziniana*) and borer (*Hedypathes betulinus*); leaf fall during periods of heavy rains; light or shadow measured with a photometer, not yet routinely incorporated to this project. These numerical data are of very limited scope, for example, it cannot be inferred from numerical data alone if a lower production in kg is due to smaller plants and fewer sprouts or to plants with greater growth and general health, but with lighter leaves. It is not possible to infer the general state of the plants nor the quantity and geometry of the branches that make up the crowns of the trees, and so on. It is not possible to visualize the microclimate impact or the geometry of the species in their consociated evolution.

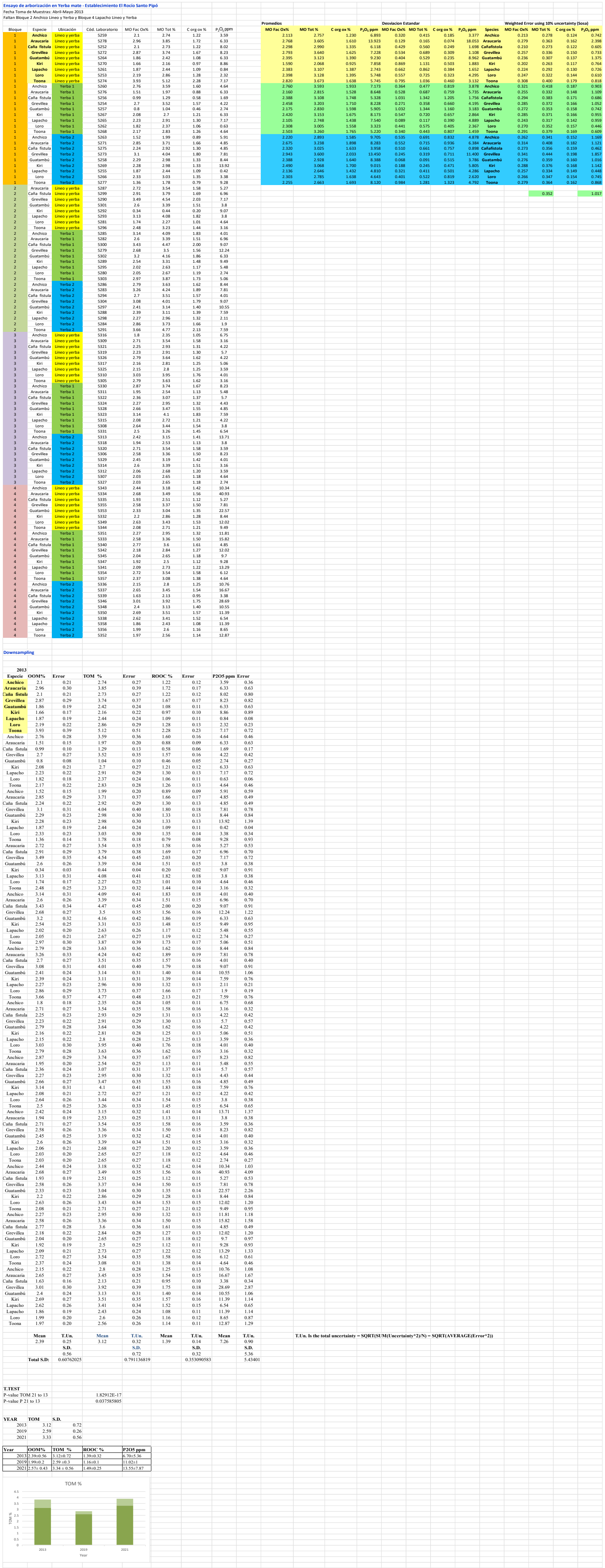

Appendix

The data sheet below is an example (soil sampling in 2013) of consolidation of the most relevant variables to communicate simple results of general interest. In yellow are soil samples from the line of *Ilex paraguariensis* plants intercalated with trees; in green from the adjacent lines at 1.5 m; in blue from the second line away from the line of trees at 3 m (in all cases the samples were taken 0.75 away from plants). We observe variations across species. Two species, Loro negro and Araucaria correlate with a decrease in SOM, a fact consistent with their narrow crowns and, in the case of Araucaria, its coniferous nature with less leafy, triangular-shaped needles[23]. These features likely reduce the litter fall compared to species with broader crowns. All other species contributed to an increase in organic matter. We also observe variations in elemental composition. For example, Toona, Anchico, and Araucaria sublots have higher nitrogen content than the rest, Lapacho, Loro negro, and Toona higher phosphorous content, followed closely by Cañafístola and Anchico while Grevilea has a lesser improvement in values and Guatambu a decrease in values[23]. While the full table of measurements is certainly of practical interest to us and INTA, repository of the data, as a guide helping us in tree species selection, this type of information is valid only locally, in this region. They could therefore subtract from the main results, which are very general. We have included here this example of soil compositional analysis with a commentary to satisfy the specialists who may have interest in more details and who may like to contact us for more information, data, and discussions.
